# External validation of machine learning models including newborn metabolomic markers for postnatal gestational age estimation in East and South-East Asian infants

**DOI:** 10.12688/gatesopenres.13131.2

**Published:** 2021-06-21

**Authors:** Steven Hawken, Malia S. Q. Murphy, Robin Ducharme, A. Brianne Bota, Lindsay A. Wilson, Wei Cheng, Ma-Am Joy Tumulak, Maria Melanie Liberty Alcausin, Ma Elouisa Reyes, Wenjuan Qiu, Beth K. Potter, Julian Little, Mark Walker, Lin Zhang, Carmencita Padilla, Pranesh Chakraborty, Kumanan Wilson

**Affiliations:** 1 Clinical Epidemiology Program, Ottawa Hospital Research Institute, Ottawa, ON, Canada; 2 School of Epidemiology and Public Health, University of Ottawa, Ottawa, ON, Canada; 3 Newborn Screening Reference Centre, University of the Philippines Manila, Manila, Philippines; 4 Pediatric Endocrinology and Genetic Metabolism, XinHua Hospital, Shanghai, Shanghai, China; 5 Better Outcomes Registry & Network, Ottawa, Canada; 6 Department of Gynecology and Obsetrics, XinHua Hospital, Shanghai, Shanghai, China; 7 MOE-Shanghai Key Lab of Children’s Environmental Health, Xinhua Hospital, Shanghai Jiao Tong University School of Medicine, Shanghai, China; 8 Department of Pediatrics, University of the Philippines Manila, Manilla, Philippines; 9 Institute of Human Genetics, National Institutes of Health, University of Philippines Manila, Manila, Philippines; 10 Newborn Screening Ontario, Children's Hospital of Eastern Ontario, Ottawa, ON, Canada; 11 Faculty of Medicine, University of Ottawa, Ottawa, ON, Canada; 12 Department of Medicine, University of Ottowa, Ottowa, ON, Canada; 13 Bruyère Research Institute, Ottowa, ON, Canada

**Keywords:** biological modelling, gestational age, preterm birth, newborn screening

## Abstract

**Background:** Postnatal gestational age (GA) algorithms derived from newborn metabolic profiles have emerged as a novel method of acquiring population-level preterm birth estimates in low resource settings. To date, model development and validation have been carried out in North American settings. Validation outside of these settings is warranted.

**Methods:** This was a retrospective database study using data from newborn screening programs in Canada, the Philippines and China. ELASTICNET machine learning models were developed to estimate GA in a cohort of infants from Canada using sex, birth weight and metabolomic markers from newborn heel prick blood samples. Final models were internally validated in an independent sample of Canadian infants, and externally validated in infant cohorts from the Philippines and China.

**Results:** Cohorts included 39,666 infants from Canada, 82,909 from the Philippines and 4,448 from China.  For the full model including sex, birth weight and metabolomic markers, GA estimates were within ±5 days of ultrasound values in the Canadian internal validation (mean absolute error (MAE) 0.71, 95% CI: 0.71, 0.72), and within ±6 days of ultrasound GA in both the Filipino (0.90 (0.90, 0.91)) and Chinese cohorts (0.89 (0.86, 0.92)). Despite the decreased accuracy in external settings, our models incorporating metabolomic markers performed better than the baseline model, which relied on sex and birth weight alone. In preterm and growth-restricted infants, the accuracy of metabolomic models was markedly higher than the baseline model.

**Conclusions:** Accuracy of metabolic GA algorithms was attenuated when applied in external settings.  Models including metabolomic markers demonstrated higher accuracy than models using sex and birth weight alone. As innovators look to take this work to scale, further investigation of modeling and data normalization techniques will be needed to improve robustness and generalizability of metabolomic GA estimates in low resource settings, where this could have the most clinical utility

## Introduction

Global- and population-level surveillance of preterm birth is challenging. Inconsistent use of international standards to define preterm birth and gestational age (GA) categories, the range of methods and timing used for GA assessment, and inadequate jurisdictional or national health data systems all hamper reliable population estimates of preterm birth ^1^. As complications related to preterm birth continue to be the most common cause of mortality for children under five ^2^, robust data on the burden of preterm birth are needed to maximize the effectiveness of resource allocation and global health interventions.

Newborn screening is a public health initiative that screens infants for rare, serious, but treatable diseases. Most of the target diseases are screened through the analysis of blood spots taken by heel-prick sampling. Samples are typically collected within the first few days after birth. Newborn samples are analyzed for a range of diseases, such as inborn errors of metabolism, hemoglobinopathies, and endocrine disorders, using tandem mass spectrometry, colorimetric and immunoassays, and high-performance liquid chromatography ^3^. Postnatal GA algorithms derived from newborn characteristics and metabolic profiles have emerged as a novel method of estimating GA after birth. Using anonymized data from state and provincial newborn screening programs, three groups in North America have developed algorithms capable of accurately estimating infant GA to within ± 1 to 2 weeks ^4– 6^. Recent work to refine metabolic GA models ^7^, as well as internally and externally validate their performance in diverse ethnic groups and in low-income settings, has demonstrated the potential of these algorithms beyond proof-of-concept applications ^8, 9^.

Published approaches to model development and validation to date have been carried out in cohorts of infants in North American settings ^4– 6^. Although internal validation of these models has been conducted among infants from diverse ethnic backgrounds ^4, 8^, external validation of model performance outside of the North American context is essential to evaluate the generalizability of models to low-income settings where they would have the most clinical utility. Birth weight, a significant covariate in all published models, is strongly correlated with GA and varies significantly by ethnicity ^10^. Metabolic variations in newborn screening profiles that result from variation in genetic and *in utero* exposures may also affect the performance of established algorithms across ethnic or geographic subpopulations ^11^. Importantly, as innovators seek to take this work to scale, validation of metabolic models using data stemming from different laboratories is warranted. In this study, we sought to validate a Canadian metabolic GA estimation algorithm in data derived from newborn screening databases based in the Philippines and China.

## Methods

### Study design

This was a retrospective cohort study involving the secondary analysis of newborn screening data from three established newborn screening programs: Newborn Screening Ontario (Ottawa, Canada); Newborn Screening Reference Centre (Manila, the Philippines); and the Shanghai Neonatal Screening Center (Shanghai, China). Approval for the study was obtained from the Ottawa Health Sciences Network Research Ethics Board (20160056-01H), and research ethics committees at both the University of the Philippines Manila (2016-269-01), and the Xinhua Hospital, Shanghai (XHEC-C-2016). The need for express informed consent from participants was waived by the ethics committees for this retrospective study.

### Study population and data sources

Newborn Screening Ontario (NSO): a provincial newborn screening program that coordinates the screening of all infants born in Ontario, Canada. The program screens approximately 145,000 infants (>99% population coverage) annually for 29 rare conditions, including metabolic and endocrine diseases, sickle cell disease, and cystic fibrosis ^12^. Newborn screening data collected between January 2012 and December 2014 were used in model building and internal validation. Further details on sample analysis methodology can be found here ^13^.

Newborn Screening Reference Center: coordinates screening across six operations sites in the Philippines. The program screens approximately 1.5 million infants (68%) annually, offering two screening panels: a basic panel of six disorders at no cost, or an expanded panel of 28 disorders paid for by families. Data from this study were obtained from one of the newborn screening centers, the National Institutes of Health at the University of the Philippines Manila. Data were included for infants born between January 2015 and October 2016 who were screened using the expanded panel of 28 disorders. Disorders screened included metabolic disorders, and hemoglobinopathies.

Shanghai Neonatal Screening Center, National Research Center for Neonatal Screening and Genetic Metabolic Diseases: coordinates the screening of infants born in Shanghai, China. The program screens approximately 110,000 infants (>98%). Four screening tests - for phenylketonuria, congenital adrenal hyperplasia, hypothyroidism and Glucose-6-phosphate dehydrogenase deficiency - are provided at no cost. Expanded newborn screening is available but must be paid for by families. Data collected from the Shanghai Jiaotong University School of Medicine Xinhua Hospital were used for this study. Infants born between February 2014 and December 2016 with expanded newborn screening results were included.

### Reference GA assessment

In newborn cohorts from Ontario and China, GA was measured using gold-standard first trimester gestational dating ultrasound in approximately 98% of cases, and was reported in weeks and days of gestation (for example 37 weeks and 6 days would be reported as 37.86 weeks). In the Philippines cohort, mothers who delivered in private hospitals generally received gestational dating ultrasounds while other infants’ GAs were generally measured using Ballard Scoring, however individual-level data identifying which GA measurement method was used was not available. GA was reported in completed weeks (for example 37 weeks and 6 days would be recorded as 37 weeks). Therefore, for the Philippines cohort only, model-based GA estimates were rounded down in the same way for comparison to reference GA in the presentation of validation results.

Specific data elements used in this study from each respective newborn screening program are provided in Table 1. All of the analytes used in our analyses were routinely collected, including quantitative fetal and adult Hb levels. The Newborn Screening Ontario (Canada) disease panel included the greatest number of analytes. All analytes included in the newborn screening panels of the Newborn Screening Reference Centre (the Philippines) and the Shanghai Newborn Screening Program (China) were also available from Newborn Screening Ontario.

**Table 1.** Newborn screening data used in model development.

Newborn Screening Ontario, Canada	Newborn Screening Reference Centre, the Philippines	Shanghai Newborn Screening Program, China
Sex, birth weight **Hemoglobins** ***F1, F, A*** **Endocrine markers** **TSH**, 17OHP **Amino Acids** alanine, arginine, citrulline, glycine, leucine, methionine, ornithine, phenylalanine, tyrosine, valine, **Acyl-carnitines, Acylcarnitine ratios** C0, C2, C3, C4, C5, C6, C8, C10, C12, C14, C16, C18, C10:1, C12:1, C14OH, C14:1, ***C14:2***, C16OH, ***C16:1OH***, ***C18OH***, C18:1, C18:2, ***C18:1OH***, C3DC, C4DC, C4OH, C5DC, C5OH, ***C5:1***, C6DC, C8:1, **Enzyme markers** ***GALT, BIO*** **Cystic fibrosis Markers** ***IRT***	Sex, birth weight **Hemoglobins** ***F1, F, A*** **Endocrine markers** TSH, 17OHP **Amino Acids** alanine, arginine, citrulline, glycine, leucine, methionine, ornithine, phenylalanine, tyrosine, valine, **Acyl-carnitines, Acylcarnitine ratios** C0, C2, C3, C4, C5, C6, C8, C10, C12, C14, C16, C18, C10:1, C12:1, C14OH, C14:1, C16OH, ***C16:1OH***, C18:1, C18:2, C3DC, C4DC, C4OH, C5DC, C5OH, C6DC, C8:1 **Enzyme markers** ***BIO*** **Cystic fibrosis Markers** ***IRT***	Sex, birth weight **Endocrine markers** TSH, 17OHP **Amino Acids** alanine, arginine, citrulline, glycine, leucine, methionine, ornithine, phenylalanine, tyrosine, valine **Acyl-carnitines, Acylcarnitine ratios** C0, C2, C3, C4, C5, C6, C8, C10, C12, C14, C16, C18, C10:1, C12:1, C14OH, C14:1, ***C14:2***, C16OH, ***C18OH***, C18:1, C18:2, C3DC, C4DC, C4OH, C5DC, C5OH, ***C5:1***, C6DC, C8:1

TSH, thyroid stimulating hormone; 17OHP, 17 hydroxyprogesterone; GALT, galactose-1-phosphate uridyl transferase; IRT, Immuno-reactive trypsinogen, BIO, biotinidase. Analytes in ***bold italics*** are not available in one or more of the external validation cohorts. Further details on the Ontario newborn screening metabolites can be found here ^15^.

### Statistical methods

***Data preparation***

Infants whose blood spot samples were collected more than 48 hours after birth were excluded from model development in the Ontario cohort. The reasons for this were twofold. First, the recommended screening sample collection window in Ontario is 24 to 48 hours after birth, and samples collected beyond that window have increasingly heterogeneous analyte results influenced by multiple exogenous factors that cannot be statistically adjusted for. And second, our GA estimation models are intended to be applied in low to middle income countries (LMICs) where samples are expected to be collected almost exclusively within the first few hours after birth.

Infants who screened positive for one or more conditions (<1%) were excluded from model development and validation in all three cohorts, which had the effect of removing a large proportion of extreme outliers and atypical metabolic profiles.

In the China and Philippines cohorts, a larger proportion of samples were collected after 48 hours so we relaxed the exclusion criteria to >72 hours, to avoid exclusion of an unacceptably large proportion of samples from the external validation cohorts. Even after relaxing the criteria, samples in preterm infants were more likely to have been collected later than 72 hours in both China and the Philippines, which resulted in an artificially lower prevalence of preterm infants in the external validation cohorts. Samples from all three cohorts were excluded if they had missing sex, birth weight, gestational age, or missing analyte values. The proportion of subjects with missing analyte or covariate data was very low so missing data imputation was not undertaken.

In preparation for use in modeling, newborn screening analytes from Ontario, the Philippines and China were winsorized using an adapted “Tukey Fence” approach ^14^. For each analyte, this involves assigning values more than three interquartile ranges above the third quartile (the upper Tukey fence) or below the first quartile (the lower Tukey fence), to the Tukey fence value. This approach preserves much of the “extremeness” of outliers but prevents extreme values from disproportionately impacting model fitting and GA estimation. The majority of measured analytes exhibit strongly right-skewed distributions, which was addressed though natural log transformation, which also stabilizes the variance, reducing the impact of heteroskedasticity. Finally, analyte levels and birth weight were normalized by subtracting the mean and dividing by the square root of the standard deviation for each variable (pareto scaling) ^16, 17^ which centers all predictors to have a mean of zero, and scales them to reduce the impact of variations in dispersion of individual analytes across cohorts. 

***Statistical modelling***

**Model Development in the Ontario, Canada model derivation cohort**

The Ontario cohort was randomly divided into three sub-cohorts: 1) a model development sub-cohort (50%); 2) an internal validation sub-cohort (25%); and 3) a test sub-cohort (25%). Stratified random sampling was used to ensure that these three sub-cohorts retained the same distribution of GA and sex as the overall cohort.

**Figure 1.**
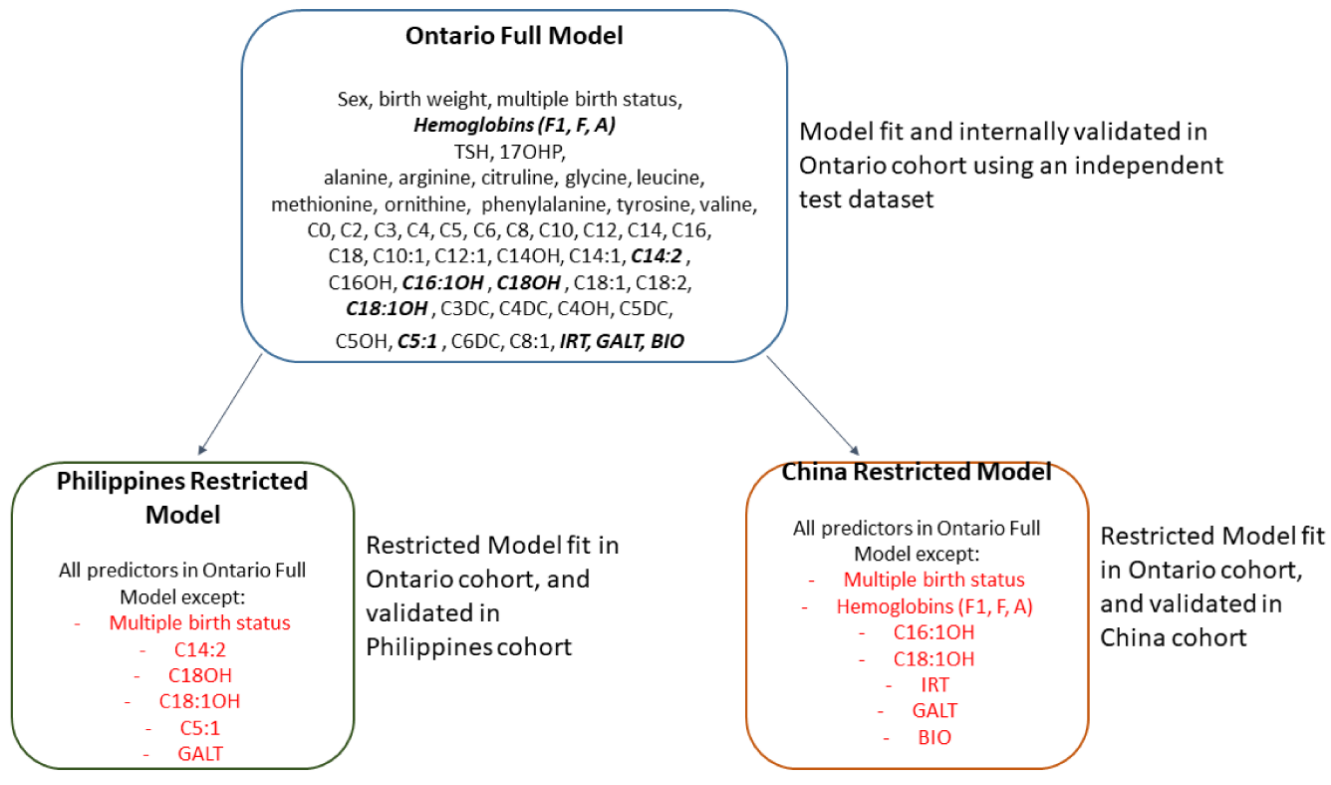
Full model vs restricted models.

A total of 47 newborn screening analytes, as well as sex, birth weight were used in the original Ontario model development. Model development included a ratio of fetal to adult hemoglobin calculated as (HbF+HbF1)/(HbF+HbF1+HbA), to measure the proportion of normal fetal hemoglobin, relative to the proportion of total normal fetal + adult hemoglobin types. GA at birth (in weeks) determined by first trimester gestational dating ultrasound was the dependent variable. A subset of screening analytes were not available in the external cohorts ( Table 1). Three main models were developed and internally validated in the Ontario cohort ( Table 2). We developed restricted models including only the covariates available in each of the two external cohorts ( Figure 1). All models were trained and internally validated within the Ontario datasets but deployed in the external cohorts.

**Table 2.** Summary of models developed and validated.

Model	Description
Model 1	Multivariable regression model including sex, birth weight and their interaction
Model 2	ELASTIC NET multivariable regression model including sex, analytes and pairwise interactions among predictors *
Model 3	ELASTIC NET multivariable regression model including sex, birth weight, analytes and pairwise interactions among * predictors *Restricted versions of Models 2 and 3 were derived based on the analyte measurements available in both the China and Philippines cohorts.

For Models 1 and 3, birth weight was the strongest predictor and was modeled using a restricted cubic spline with five knots to allow for non-linearity of the association of birth weight with gestational age. For Models 2 and 3, we included all pre-specified covariate main effects in the models, however we additionally identified the most predictive analytes using the metric of generalized partial Spearman correlation that detects non-linear and non-monotonic associations with GA, mutually adjusted for all other analytes and clinical covariates. Based on this partial Spearman correlation analysis there were seven analyte covariates that had distinctly stronger partial correlations with GA compared to all others. These seven analytes were modeled using restricted cubic splines with 5 knots. These were (in order of strength of partial spearman correlation): fetal-to-adult hemoglobin ratio, 17-OHP, C4DC, TYR, ALA, C5, and C5DC. For birthweight, and the seven strongest analyte predictors, knot placement was at the 5th, 27.5th, 50th, 72.5th, and 95th percentiles based on the Ontario population distribution of these analytes ^18^. Hemoglobin measurements were unavailable in the China cohort, so the China restricted models included restricted cubic splines for the other top six analytes.

For Model 1, all covariates and pairwise interactions were included in the model without variable selection or regularization. For Models 2 and 3 we employed Elastic Net regularization, which employs two forms of penalization (called L1 and L2 regularization) to simultaneously estimate regression coefficients while also shrinking them towards zero to penalize the increase in model complexity from each additional term included in the model ^19^. The Elastic Net regression methodology allows models to be fit with a large number of predictors, and allows models to be fit even in situations where models where the number of predictors exceeds the number of observations (p>>n). This regularization strategy provides strong protection against overfitting, and against the instability inherent in fitting models with a large number of predictors relative to the number of observations available for model fitting ^19, 20^. 

**Validation of GA estimation models**

We applied the identical approach in validating models internally (in Ontario test cohort) and externally (China and Philippines cohorts). Final regression model equations derived from the Ontario model development sub-cohort were used to calculate an estimated gestational age in the independent Ontario test cohort and in the China and Philippines cohorts. Model accuracy metrics were based on residual errors: the difference between model-estimated GA and reference GA. Although mean square error (MSE) is typically the loss function used in maximum-likelihood model fitting for continuous outcomes, it is not necessarily the best metric for assessing average agreement in model validation, as it is based on sum of squared differences, and hence is sensitive to large and small residuals. Therefore, the primary metric we have presented is the mean absolute error (MAE). MAE is the average of absolute values of residuals (values of the model estimate minus the reference GA) across all observations. MAE reflects the average deviation of the model estimate compared to the reference estimate, expressed in the same units as GA (weeks).

$$MAE=\frac{1}{n}\sum_{i=1}^{n}\left|GA_{ref_{i}}-GA_{est_{i}}\right|$$

For completeness, as well as for comparability to other published validations, we also report the square root of the MSE (RMSE). Also known as the standard error of estimation, RMSE is also expressed in the same units as GA (weeks).

$$RMSE=\sqrt{\frac{1}{n}\sum_{i=1}^{n}{\left(GA_{ref_{i}}-GA_{est_{i}}\right)}^{2}.}$$

Lower values of both MAE and RMSE reflects more accurate model estimated GA. For example, a reported MAE of 1.0 week reflects that the average discrepancy between model estimated GA and reference GA was 7 days. We also calculated the percentage of infants with GAs correctly estimated within ±7 and ±14 days of reference GA. We assessed model performance overall and in important subgroups: preterm birth (<37 weeks GA), and small-for-gestational age: below the 10th (SGA10) and 3rd (SGA3) percentile for birth weight within categories of gestational week at delivery and infant sex based on INTERGROWTH-21 gestational weight for GA percentiles ^21^. Parametric standard error estimates were not readily calculable for our performance metrics, therefore we calculated 95% bootstrap percentile confidence intervals based on the 2.5th and 97.5th percentiles over 1000 bootstrap replicates for each validation cohort ^22^. Replication code is available as Extended data ^23^. To assess the overall calibration of our models we produced residual plots to visually assess accuracy across the spectrum of observed and estimated GA. To assess calibration in the large, we estimated the overall preterm birth rate using estimated GA derived from each model and compared this to the true preterm birth rate based on observed GA in each validation cohort.

## Results

### Cohort characteristics

Cohort characteristics are presented in Table 3. In all, the final infant cohorts for model validation included 39,666 infants from Ontario, Canada, 82,909 infants from the Manila, Philippines cohort and 4,448 infants from the Shanghai, China cohort. Mean (SD) of clinically reported GAs for the Ontarian, Filipino and Chinese cohorts were 39.3 (1.6), 38.5 (1.4) and 38.9 (1.4) weeks, respectively. Preterm infants (GA <37 weeks) comprised 2,226/39,666 (5.6%) of the Ontario cohort, 3,832/82,909 (4.6%) of the Philippines cohort, and 215/4,448 (4.8%) of the China cohort.

**Table 3.** Cohort Characteristics.

	Canada n=39,666 (Ontario test cohort)	Philippines n=82,909	China n=4,448
**Sex, n (%)**			
Male	19,536 (49.3%)	42,867 (51.7%)	2,351 (52.9 %)
Female	20,130 (50.7%)	40,042 (48.3%)	2,097 (47.1 %)
**Birth weight (g), mean±SD**			
Overall	3,379 ± 530	3,001 ± 452	3,337 ± 437
Term infants only	3,431 ± 476	3,044 ± 414	3,369 ± 407
Preterm infants only	2,504 ± 623	2,250 ± 539	2,710 ± 536
Low birth weight (<2500g), n (%)	1812 (4.6%)	8423 (10.2%)	128 (2.9%)
SGA (<10 th Centile), n (%)	1,561 (3.9%)	11,295 (13.6%)	123 (2.8%)
SGA (<3 rd Centile), n (%)	363 (0.9%)	3,407 (4.1%)	19 (0.4 %)
LGA (>90 th Centile), n (%)	8734 (22.0%)	4780 (5.8%)	741 (16.7%)
Completed gestational age (wks), mean±SD	39.3±1.6	38.5±1.4	38.9±1.4
Term (≥37 wks), n (%)	37,440 (94.4%)	79,077 (95.4%)	4,233 (95.2%)
Late Preterm (32–36 wks), n (%)	2,049 (5.2%)	3,566 (4.3%)	197 (4.4 %)
Very Preterm (28–31 wks), n (%)	126 (0.3%)	233 (0.3%)	11 (0.3 %)
Extremely Preterm (<28 wks), n (%)	51 (0.1%)	33 (0.0%)	7 (0.2 %)

SGA, small for gestational age (lowest 10 and 3 centiles within gestational age and sex strata, calculated in the Ontario cohort using Intergrowth-21 centiles and applied uniformly in the Ontario, China and Philippines cohorts)

### Model performance in Ontario, Canada

The most predictive individual analytes in Models 2 and 3 were fetal-to-adult hemoglobin ratio, 17-OHP, C4DC, TYR, ALA, C5, and C5DC. Modeling this subset of analytes using restricted cubic splines further increased their predictive power. Sensitivity analyses were conducted modeling the most predictive analytes using linear terms versus restricted cubic splines, as well a sensitivity analysis comparing models developed with only main effects versus allowing pairwise interactions, and in both cases model performance in terms of MAE/RMSE and adjusted R-square was markedly improved. Therefore, final models included splines and pairwise interactions.

 Estimation of GA using Model 1 (including only sex and birth weight) yielded an MAE (95% CI) of 0.96 (0.96, 0.97) weeks in the Ontario cohort, indicating that the model provided GA estimates that were accurate to within ± 7 days of reference GA. Model 2, (including sex and metabolomic markers), was accurate within an average of ± 6 days (MAE 0.79 (0.79, 0.80) weeks). Model 3, which included sex, birth weight and metabolomic markers was the most accurate, estimating GA within about ± 5 days of ultrasound-assigned GA (MAE 0.71 (0.71, 0.72) weeks), and estimated GA within ± 1 week in 74.6% of infants overall. Model 3 was the best performing model in preterm infants (GA<37 weeks), with an MAE (95% CI) of 1.03 (0.99, 1.06) compared to MAE of 1.78 (1.73, 1.82) for Model 1 and 1.25 (1.21, 1.29) for Model 2. In contrast, Model 2, which did not include birth weight, performed the best in growth restricted infants, with MAE of 0.90 (0.85 to 0.94) in SGA10 infants and 1.03 (0.92, 1.13) in SGA3 infants, and was slightly better than Model 3, which did include birth weight. However, Model 1, including only sex and birth weight, was extremely inaccurate in both SGA10 and SGA3 infants with MAE of 2.71 (2.66, 2.76) and 3.84 (3.75, 3.95) respectively ( Table 4).

**Table 4.** Summary of model performance to estimate gestational age.

Models	Ontario Cohort				Philippines Cohort				China Cohort			
	Overall, N=39,666	<37 weeks, N=2,226	SGA10, N=1561	SGA3, N=363	Overall, N=82,909	<37 weeks, N=3828	SGA10, N=11,294	SGA3, N=3407	Overall, N=4448	<37 weeks, N=215	SGA10, N=123	SGA3, N=19
*Model 1: Sex and Birth weight*												
*MAE (CI)* *RMSE (CI)* *% +/-1 wk (CI)* * % +/- 2 wks (CI)*	0.96 (0.96, 0.97) 1.23 (1.22, 1.24) 60.0 (59.5, 60.5) 90.9 (90.6, 91.2)	1.78 (1.73, 1.82) 2.13 (2.07, 2.18) 30.2 (28.5, 32.0) 60.5 (58.4, 62.6)	2.71 (2.66, 2.76) 2.86 (2.80, 2.91) 0.4 (0.1, 0.7) 21.7 (19.5, 23.8)	3.84 (3.75, 3.95) 3.96 (3.86, 4.06) 0.6 (0.0, 1.4) 1.1 (0.3, 2.3)	0.96 (0.95, 0.97) 1.30 (1.29, 1.31) 78.2 (78.0, 78.5) 95.5 (95.3, 95.6)	1.87 (1.83, 1.92) 2.31 (2.24, 2.37) 43.4 (42.0, 45.0) 68.9 (67.4, 70.4)	1.47 (1.45, 1.49) 1.83 (1.80, 1.86) 61.0 (60.1, 61.8) 85.4 (84.7, 86.0)	2.65 (2.62, 2.69) 2.84 (2.80, 2.89) 5.0 (4.3, 5,8) 53.6 (51.8, 55.3)	0.90 (0.87, 0.92) 1.23 (1.17, 1.29) 65.5 (64.1, 66.9) 91.7 (90.8, 92.5)	2.02 (1.76, 2.33) 2.85 (2.37, 3.39) 33.4 (27.2, 39.7) 58.6 (52.1, 65.2)	2.72 (2.60, 2.85) 2.82 (2.68, 2.96) 0.0 (0.0, 0.0) 13.7 (7.7, 20.2)	3.90 (3.66, 4.15) 3.93 (3.69, 4.19) 0.0 (0.0, 0.0) 0.0 (0.0, 0.0)
*Model 2: Sex and Analytes*												
**Ontario** *MAE (CI)* *RMSE (CI)* *% +/-1 wk (CI)* * % +/- 2 wks (CI)*	0.79 (0.79, 0.80) 1.02 (1.01, 1.03) 69.4 (69.0, 69.9) 95.1 (94.9, 95.4)	1.25 (1.21, 1.29) 1.57 (1.51,1.62) 46.9 (45.0, 48.9) 80.3 (78.8, 82.0)	0.90 (0.85, 0.94) 1.19 (1.13, 1.26) 66.6 (64.0, 69.0) 91.7 (90.3, 93.1)	1.03 (0.92, 1.13) 1.42 (1.27, 1.59) 61.9 (56.9, 67.1) 88.1 (84.6, 91.6)	-	-	-	-	-	-	-	-
**Philippines-** **restricted** *MAE (CI)* *RMSE (CI)* *% +/-1 wk (CI)* *% +/- 2 wks (CI)*	0.81 (0.80, 0.81) 1.04 (1.03, 1.05) 68.5 (68.1, 69.0) 94.8 (94.6, 95.0)	1.28 (1.24, 1.32) 1.59 (1.54, 1.64) 45.4 (43.4, 47.4) 80.0 (78.3, 81.7)	0.92 (0.89, 0.97) 1.22 (1.16, 1.28) 63.8 (61.4, 66.1) 91.2 (89.7, 92.6)	1.04 (0.93, 1.14) 1.42 (1.26, 1.57) 61.6 (56.2, 66.7) 88.1 (84.6, 91.5)	1.02 (1.02, 1.03) 1.37 (1.36, 1.37) 75.3 (75.1, 75.6) 94.3 (94.1, 94.5)	1.96 (1.91, 2.01) 2.43 (2.37, 2.50) 41.93 (40.3, 43.5) 69.3 (67.8, 70.7)	1.08 (1.06, 1.09) 1.44 (1.42, 1.47) 73.1 (72.3, 73.9) 92.3 (91.8, 92.8)	1.18 (1.14, 1.2) 1.57 (1.53, 1.62) 70.0 (68.7, 71.5) 89.8 (88.8, 90.7)	-	-	-	-
**China-** **restricted** *MAE (CI)* *RMSE (CI)* *% +/-1 wk (CI)* *% +/- 2 wks (CI)*	0.87 (0.87, 0.88) 1.12 (1.11, 1,13) 64.9 (64.5, 65.) 93.0 (92.8, 93.3)	1.48 (1.44, 1.52) 1.80 (1.75, 1.85) 39.8 (37.9, 42.0) 71.8 (70.1, 73.8)	0.98 (0.94, 1.03) 1.31 (1.25, 1.38) 60.9 (58.3, 63.4) 89.0 (87.3, 90.6)	1.13 (1.03, 1.25) 1.58 (1.40, 1.76) 57.1 (51.6, 62.3) 83.2 (79.1, 87.3)	-	-	-	-	1.07 (1.04, 1.10) 1.44 (1.38, 1.52) 56.2 (54.7, 57.6) 86.8 (85.9, 87.8)	2.49 (2.21, 2.80) 3.32 (2.80, 3.86) 16.8 (11.9, 21.8) 46.9 (40.3, 53.3)	1.00 (0.84, 1.15) 1.34 (1.15, 1.52) 62.0 (53.4, 70.9) 86.1 (79.8, 91.7)	1.03 (0.63, 1.45) 1.36 (0.84, 1.92) 58.9 (36.4, 82.4) 89.3 (74.5, 100.0)
*Model 3: Sex, Birth weight and Analytes*												
**Ontario** *MAE (CI)* *RMSE (CI)* *% +/-1 wk (CI)* *% +/- 2 wks (CI)*	0.71 (0.71, 0.72) 0.92 (0.91, 0.93) 74.6 (74.2, 75.1) 97.0 (96.8, 97.2)	1.03 (0.99, 1.06) 1.32 (1.27, 1.37) 58.0 (55.9, 60.2) 88.8 (87.5, 90.3)	1.13 (1.09, 1.17) 1.42 (1.36, 1.47) 50.5 (48.2, 53.0) 85.0 (83.2, 86.6)	1.48 (1.37, 1.60) 1.81 (1.67, 1.96) 35.8 (30.6, 40.6) 73.8 (69.1, 78.1)	-	-	-	-	-	-	-	-
**Philippines-** **restricted** *MAE (CI)* *RMSE (CI)* *% +/-1 wk (CI)* *% +/- 2 wks (CI)*	0.72 (0.71, 0.72) 0.92 (0.91, 0.93) 74.5 (74.1, 74.9) 69.9 (96.8, 97.1)	1.04 (1.00, 1.07) 1.33 (1.28, 1.38) 57.0 (54.9, 59.0) 88.2 (86.8, 89.6)	1.15 (1.10, 1.19) 1.43 (1.37, 1.49) 50.1 (47.6, 52.8) 85.0 (83.2, 86.8)	1.49 (1.38, 1.60) 1.82 (1.68, 1.97) 35.2 (30.3, 39.8) 73.5 (68.5, 77.8)	0.90 (0.90, 0.91) 1.21 (1.20, 1.22) 80.4 (80.1, 80.7) 97.0 (96.8, 97.1)	1.49 (1.45, 1.53) 1.92 (1.86, 1.98) 57.2 (55.8, 58.6) 84.1 (82.8, 85.3)	0.97 (0.96, 0.99) 1.31 (1.29, 1.33) 77.4 (76.5, 78.2) 94.7 (94.2, 95.1)	1.27 (1.23, 1.3) 1.63 (1.60, 1.67) 65.1 (63.5, 66.6) 88.3 (87.2, 89.3)	-	-	-	-
**China-** **restricted** *MAE (CI)* *RMSE (CI)* *% +/-1 wk (CI)* *% +/- 2 wks (CI)*	0.76 (0.75, 0.76) 0.97 (0.96, 0.98) 71.8 (71.3, 72.2) 96.3 (96.1, 96.5)	1.12 (1.08, 1.15) 1.43 (1.37, 1.48) 53.1 (50.1, 55.2) 85.1 (83.5, 86.7)	1.26 (1.21, 1.31) 1.54 (1.48, 1.59) 44.1 (41.7, 46.6) 83.2 (81.2, 85.1)	1.65 (1.54, 1.78) 1.98 (1.84, 2.13) 29.4 (24.7, 34.2) 69.9 (64.8, 74.9)	-	-	-	-	0.89 (0.86, 0.91) 1.20 (1.14, 1.27) 64.7 (63.3, 66.0) 92.7 (92.0, 93.4)	1.74 (1.49, 2.05) 2.69 (2.16, 3.28) 43.4 (36.9, 50.2) 72.1 (66.2, 78.0)	1.48 (1.32, 1.64) 1.70 (1.54, 1.88) 30.9 (22.7, 39.6) 77.3 (69.3, 84.7)	2.04 (1.68, 2.45) 2.19 (1.78, 2.65) 4.9 (0.0, 16.7) 47.2 (25.0, 70.7)

Data are presented as the mean and 2.5 ^th^ and 97.5 ^th^ bootstrap percentiles for MAE, RMSE and the percentage of model estimates within 1 and 2 weeks of ultrasound GA for 1000 bootstrap samples generated from each cohort

Restricted models including the subset of analytes available in the Philippines and China cohorts performed comparably to the unrestricted Ontario models overall. When applied to the Ontario internal validation cohort, accuracy of both the China- and Philippines-restricted models was slightly lower overall and lower in important subgroups, most notably in preterm and growth restricted infants for cohort (Model 2 and Model 3 China restricted and Philippines restricted) ( Table 4).

### External validation of model performance in the Philippines cohort

When applied to infant samples from the Philippines cohort, Model 1 yielded a MAE (95% CI) of 0.96 (0.95, 0.97). Accuracy was slightly decreased for Model 2, with MAE of 1.02 (1.02, 1.03). Model 3 which included sex, birth weight and screening analytes available in the Philippines database performed the best, with an MAE of 0.90 (0.90, 0.91). Model 3 was also the best performing model in preterm infants, with MAE of 1.49 (1.45, 1.53) compared to 1.87 (1.83, 1.92) for Model 1 and 1.96 (1.91, 2.01) for Model 2. Model 3 also yielded the most accurate GA estimates in growth restricted infants, with MAE of 0.97/1.27 for SGA10/SGA3 infants compared to 1.47/2.65 for Model 1 and 1.08/1.18 for Model 2 for SGA10/SGA3 infants ( Table 4).

Based on GA estimates from Model 3, the estimated preterm birth prevalence was 4.2% (95% CI: 4.1%, 4.4%), compared to the true prevalence of 4.3% using reference GA in the Philippines cohort. Both Model 1 and Model 2 overestimated the preterm birth rate, at 5.1% (4.9%, 5.4%) and 5.0%(4.9%, 5.2%), respectively.

### External validation of model performance in the China cohort

In the China cohort, Model 1 estimated GA to within 6 days overall, with an MAE of 0.90 (0.87, 0.92). Model 3 demonstrated similar accuracy to Model 1 with MAE of 0.89 (0.86, 0.91), and Model 2 performed the worst with MAE of 1.07 (1.04, 1.10). Model 3 performed the best in preterm infants, with MAE of 1.74 (1.49, 2.05) versus 2.49 (2.21, 2.80) for Model 2 and 2.02 (1.76, 2.33) for Model 1. In growth restricted infants, Model 2 was the most accurate, with MAE of 1.00/1.03 in SGA10/SGA3 infants compared to 1.48/2.04 for Model 3 and 2.72/3.90 for Model 1.

Based on GA estimates from Model 3, the estimated preterm birth prevalence was 4.2% (95% CI: 3.7%, 4.8%), and Model 1, which demonstrated similar overall accuracy, estimated a rate of 4.9% (4.3%, 5.6%). Model 2, the least accurate of the three in the China cohort, underestimated the preterm birth rate to be 3.6% (2.9%,4.3%), compared to the actual preterm birth rate of 4.8% based on reference GA in the China cohort.

### Model performance across spectrum of GA

**Figure 2.**
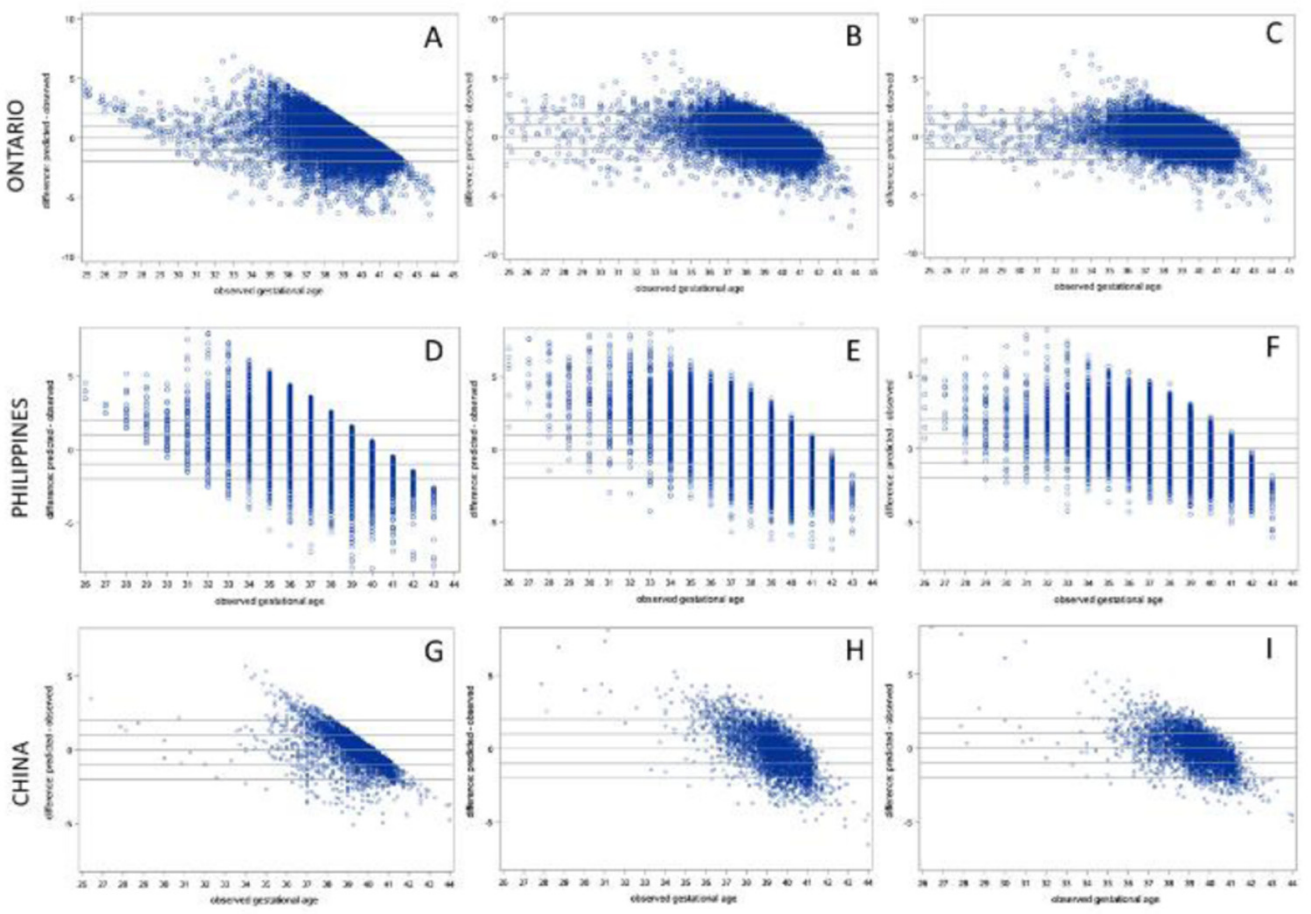
Residual plots of predicted – observed by ultrasound-assigned gestational age. Models applied to test cohort from Ontario: ( **A**) Model 1 (sex + birthweight), ( **B**) Model 2 (sex + analytes) ( **C**) Model 3 (sex + birthweight +analytes). Models applied to cohort from the Philippines: ( **D**) Model 1, ( **E**) Model 2, and ( **F**) Model 3. Models applied to cohort from China: ( **G**) Model 1, ( **H**) Model 2, and ( **I**) Model 3.

**Figure 3.**
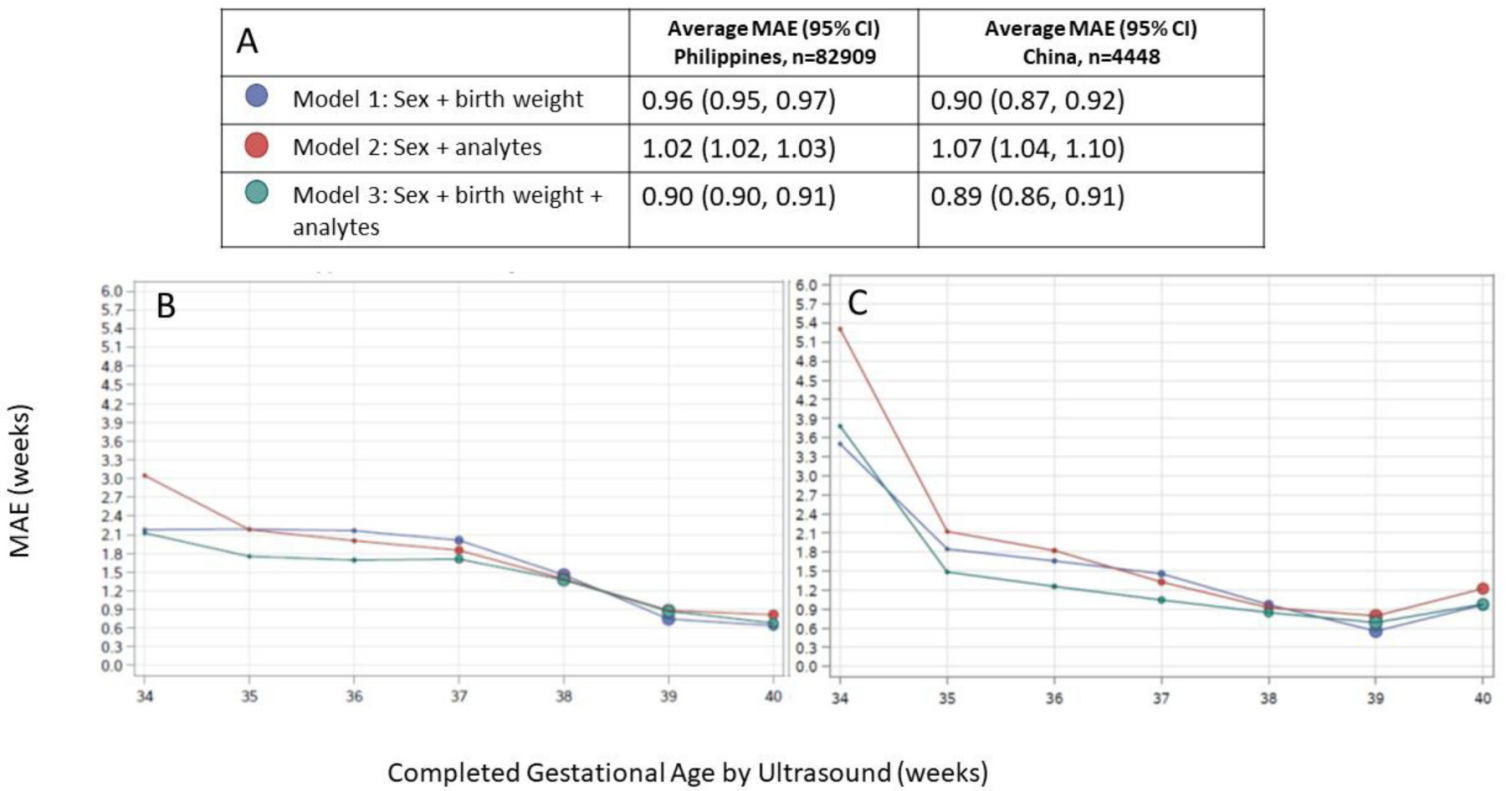
Agreement between algorithmic gestational age estimations compared to ultrasound-assigned gestational age. ( **A**) Legend, and overall MAE (95% CI) for each model applied to data from the Philippines and China. Dot size in plots is proportional to sample size in each gestational age category. Performance of each model by ultrasound-assigned gestational age when applied to data from ( **B**) the Philippines ( **C**) China. MAE, mean absolute error (average absolute deviation of observed vs. predicted gestational age in weeks).

Scatter plots of observed GA versus estimated GA for all three models in the Ontario, Philippines and China cohorts are presented in Figure 2, which shows that in general, lower (preterm) GAs tend to be overestimated by all three models when applied to all cohorts. In all models applied to both external validation cohorts, GA estimates were most accurate in term infants and accuracy tended to be lower in preterm infants. Across the spectrum of ultrasound-assigned GA, Model 3 provided the most accurate estimates overall Figure 3).

## Discussion

In this study, we demonstrated that the performance of gestational dating algorithms developed in a cohort of infants from Ontario, Canada including newborn screening metabolomic markers from dried blood-spot samples was attenuated when the models were applied to data derived from external laboratories and populations. When these Canadian-based models were tailored to the analytes available from newborn screening programs in Shanghai, China and Manila, Philippines, the models were less accurate in estimating absolute GA in infant cohorts from these locations than when the same models were applied to an Ontario infant cohort. Models including analytes generally demonstrated improved accuracy over those relying on sex and birth weight alone, but the added benefit of models including blood-spot metabolomic markers (Model 2 and Model 3) was not substantial when looking at overall accuracy. However, our models that included metabolomic markers did demonstrate markedly improved accuracy over sex and birth weight in important subgroups (preterm and growth restricted infants), with Model 3 which included sex, birth weight and metabolomic markers demonstrating the best performance in almost all settings. The exception to this observation was in growth restricted infants (SGA10 and SGA3), where Model 2 often performed the best. This is not surprising, as birth weight is clearly a misleading predictor of GA in growth restricted infants, and although Model 3 still outperformed Model 1, its accuracy was impacted by the inclusion of birth weight in addition to metabolomic markers. Therefore, the decision of whether to prefer Model 2 or Model 3 may hinge on whether the prevalence of growth restriction is known to be high in the setting where the GA estimation algorithm is to be deployed. When we compared preterm birth rates (<37 weeks GA) calculated based on model estimates, to those calculated based on reference GA in each cohort, the model-based estimates from the best performing model (Model 3) agreed reasonably well with the reference preterm birth rates (4.2% vs 4.8% for China and 4.2% vs 4.6% for the Philippines). Unfortunately, as with any dichotomization of a continuous measure (GA), there are significant edge effects that can contribute to perceived misclassification (e.g. GA of 36.9 weeks is classified as preterm while a GA of 37.1 weeks is classified as term, despite a difference in GA of only about 1 day).

There are several reasons why the metabolic gestational dating algorithm we developed from a North American newborn cohort may not have performed as well using data derived from other infant populations. First, as observed in the differences in Model 1’s performance across the three cohorts, the predictive utility of anthropomorphic measurements for estimating GA may vary across populations. Second, metabolic profiles may be influenced by the differences in genetic and environmental exposures experienced by each cohort. Previous validation of our models among infants born in Ontario to landed-immigrant mothers from eight different countries across Asia and North and Sub-Saharan Africa suggested that inherent biological differences may not be a significant contributor to newborn metabolic data and the performance of our algorithms ^8^. However in an external validation of previously developed GA estimation models in a prospective cohort from South Asia ^9^, the drop in performance was more pronounced, despite the centralized analysis of samples in the Ontario Newborn Screening lab. Third, variations in the clinical measures of GA used across the cohorts may have impeded the accuracy of our algorithms. Our GA models were developed with first trimester ultrasound-assigned GA as the dependent variable. Whereas first trimester ultrasounds were the gold standard in the Ontario and China cohorts, GAs for the Philippines cohort were determined by a mixture of gestational dating ultrasound and Ballard scores, and were only available to the nearest completed week of GA. Ballard tends to overestimate gestational age with a wide margin of error, particularly in preterm infants ^24^. Lastly, and perhaps most importantly, variations in the collection procedures and analytical methods used by each of the newborn screening programs are likely to have impacted the measurable relationship between the analytes and newborn GA. At the newborn screening program in Shanghai, China, samples were collected, on average, about one day later than samples used for model development, particularly among preterm infants with the majority being collected between 48–72 hours. Variations in temperature, climate, sample handling, and storage among the three newborn screening laboratories may have also contributed to heterogeneity of findings. The screening laboratories in Ontario, Shanghai, China and Manila also varied with respect to sample collection and handling, as well as lab equipment, assays and reagents to quantify the measured analytes. Reduced performance of models in the China and Philippines settings was likely due to a combination of these sources of heterogeneity including genetic, environmental and non-biological (eg laboratory-based) variation, which cannot easily be teased apart. We attempted to address these sources of heterogeneity and bias through our data preparation steps, which involved local standardization of analyte values and birth weight. Extreme outliers, skewed distributions, heteroscedasticity, and systematic biases within and between laboratories are all factors that may obscure biological signals. Normalization and other data pre-processing steps are therefore crucial to the analysis of metabolomic data, and we continue to investigate the impact of alternative data normalization techniques in improving the generalizability of our GA estimation models, while still taking care to preserve the biological signals of interest. This is an active area of active research as it relates to the use of ‘omics data in prognostic models more generally ^25, 26^.

Our study has several strengths and limitations. Notable strengths include the size of our Ontario, China and Philippines cohorts, the commonality of a preponderance of the analytes across populations, the ability to tailor models to the specific analytes available for each cohort, and the methodological rigor we imposed in our modeling and validation. Limitations include the inability to examine the impact of environmental factors (socio-economic conditions, dietary and environmental exposures during pregnancy), variations in approaches to newborn screening that may not have been accounted for in our analyses, and generally smaller sample sizes for more severely preterm children. The preterm birth rate in our external validation cohorts (Philippines:4.6%, China: 4.8%) was lower than published estimates (Philippines: 15%, China: 7.1%) ^27^. This was in part due to selection bias because the expanded screening panel was not universally covered, requiring the family to pay in many cases, which may have led to infants in our validation cohorts being more likely to be from affluent families and/or from urban versus rural areas. Another source of bias was that samples in infants born preterm in China, and to a lesser extent the Philippines, were much more likely to be collected later than in term infants, and often after 48-72 hours. Samples collected beyond 48 hours were more heterogenous than samples collected within 48 hours but excluding these would have excluded an unacceptably large proportion of infants overall, especially preterm infants. Our compromise approach of relaxing the criteria to exclude samples that were collected later than 72 hours after birth, included more infants at the cost of increased heterogeneity, but even so, still excluded a disproportionate number of preterm infants in the China and Philippines. A combination of these factors likely contributed to the lower than expected preterm birth rates observed in China and in the Philippines validation cohorts, as well as leading to decreased apparent model performance due to more heterogeneous samples (collected 48-72 hours after birth) being included.

While there are numerous options currently available to health care providers to determine postnatal GA, none are as accurate as first trimester dating ultrasound ^28^. Where access to antenatal dating technologies are limited, and the reliability of postnatal assessments is variable, there is a recognized need for new and innovative approaches to ascertaining population-level burdens of preterm birth in low resource settings ^28, 29^. Metabolic GA estimation models in particular have proven particularly promising ^29^, and we continue to refine and evaluate these models in a variety of populations ^6, 7, 15^ and laboratories in an effort to ready this innovation for broader application. The findings of this study suggest that the accuracy of metabolic gestational dating algorithms may be improved where newborn samples can be analyzed in the same laboratories from which the algorithms were originally derived and underscore our previous findings of their potential particularly among low birth weight or SGA infants ^7^. Validation of our ELASTIC NET machine learning models is also being completed in prospective cohorts of infants from low-income settings in Bangladesh and Zambia ^15^, with validation of previously developed models already completed in Bangladesh ^9^. The effects of laboratory-specific variables are being mitigated through the standardization of collection and analytical procedures applied to newborn samples; preliminary results are promising. As efforts to optimize gestational dating algorithms based on newborn metabolic data continue, and innovators seek to take this work to scale, future work should identify opportunities to develop algorithms locally where newborn screening laboratories exist, and to build capacity in low resource settings for these purposes.

## Data availability

### Underlying data

The data from Ontario, Canada used to develop models, and the data for the external validation cohorts in which model performance was evaluated were obtained through bilateral data sharing agreements with the Ontario Newborn Screening Program and BORN Ontario, and newborn screening laboratories at Xinhua Hospital in Shanghai, China and University of the Philippines, Manila, Philippines. These data sharing agreements prohibited the sharing of patient-level data beyond our research team.

### Ontario data

Those wishing to request access to Ontario screening data can contact newbornscreening@cheo.on.ca, and the request will be assessed as per NSO’s data request and secondary use policies. For more information, please visit the NSO website: https://www.newbornscreening.on.ca/en/screening-facts/screening-faq (‘What happens when a researcher wants to access stored samples for research’); https://www.newbornscreening.on.ca/en/privacy-and-confidentiality.

### Philippines data

Researchers can request access to the de-identified data (sex, birthweight, gestational age and screening analyte levels) from the Philippines for future replication of the study by sending a request letter to the Director of Newborn Screening Reference Center stating the study objectives in addition to:

a. A copy of the study protocol approved by a technical and ethics review board that includes methods and statistical analysis plans;
b. Full name, designation, affiliation of the person with whom the data will be shared; and,
c. Time period that the data will be accessed.

Data requests must be addressed to: Dr. Noel R. Juban, Director of the Newborn Screening Reference Center National Institutes of Health, Unit 304 New Gold Bond Building, 1579 F. T. Benitez St, Ermita, Manila, Philippines, info@newbornscreening.ph.

China Data

Researchers can request access to the de-identified data (sex, birthweight, gestational age, age at sample collection, and screening analyte levels) from China by sending a written request to the corresponding author, Dr. Steven Hawken ( shawken@ohri.ca), which must include a copy of the study protocol and approval from the researcher’s local ethics board.

### Extended data

SAS and R code for data preparation and cleaning, model fitting and external model validation are available at: https://github.com/stevenhawken/Gates-Repository-China-Phil.

Archived code at time of publication: http://doi.org/10.5281/zenodo.4085320 ^23^.

License: GNU General Public License v3.
