# Supplementary material for: External validation of machine learning models including newborn metabolomic markers for postnatal gestational age estimation in East and South-East Asian infants
Source: Gates Open Res. 2021 Jun 21;4:164. Originally published 2020 Oct 29. [Version 2] doi: 10.12688/gatesopenres.13131.2 (PMC8160452; doi:10.12688/gatesopenres.13131.2)
Supplement: Peer review history

# Peer review: Reviewer response for version 2

**DOI:** 10.21956/gatesopenres.14541.r30793

**Published:** 2021-07-01

**Authors:** Julie Courraud

**Affiliations:** 1 Section for Clinical Mass Spectrometry, Statens Serum Institut, Copenhagen, Denmark

The authors have addressed my comments appropriately.

Is the work clearly and accurately presented and does it cite the current literature?

Yes

If applicable, is the statistical analysis and its interpretation appropriate?

I cannot comment. A qualified statistician is required.

Are all the source data underlying the results available to ensure full reproducibility?

No

Is the study design appropriate and is the work technically sound?

Yes

Are the conclusions drawn adequately supported by the results?

Yes

Are sufficient details of methods and analysis provided to allow replication by others?

Partly

Reviewer Expertise:

Metabolomics, LC-MS/MS, clinical study design, clinical assays and quality

I confirm that I have read this submission and believe that I have an appropriate level of expertise to confirm that it is of an acceptable scientific standard.

# Peer review: Reviewer response for version 2

**DOI:** 10.21956/gatesopenres.14541.r30796

**Published:** 2021-06-30

**Authors:** José Villar, Eric Ohuma

**Affiliations:** 1 Nuffield Department of Women's and Reproductive Health, University of Oxford, Oxford, UK; 2 London School of Hygiene & Tropical Medicine, London, UK

Thank you to the authors for taking time to address the previous comments and subsequent changes to the manuscript. No further comments

Is the work clearly and accurately presented and does it cite the current literature?

Yes

If applicable, is the statistical analysis and its interpretation appropriate?

Partly

Are all the source data underlying the results available to ensure full reproducibility?

Yes

Is the study design appropriate and is the work technically sound?

Yes

Are the conclusions drawn adequately supported by the results?

Partly

Are sufficient details of methods and analysis provided to allow replication by others?

Yes

Reviewer Expertise:

Medical Statistician with experience in the field of maternal and child health

We confirm that we have read this submission and believe that we have an appropriate level of expertise to confirm that it is of an acceptable scientific standard.

# Peer review: Reviewer response for version 1

**DOI:** 10.21956/gatesopenres.14318.r30339

**Published:** 2021-05-27

**Authors:** Ramesh Agarwal, Suman Chaurasia

**Affiliations:** 1 Newborn Health Knowledge Centre, WHO Collaborating Centre For Training and Research in Neonatal Care, Department of Pediatrics, All India Institute of Medical Sciences, New Delhi, Delhi, India; 2 Department of Neonatology, All India Institute of Medical Sciences (AIIMS), Rishikesh, Uttarakhand, India

*Authors: Suman Chaurasia, Ramesh Agarwal*

- We are pleased to go through this interesting article by Hawken *et al.*, dealing with estimation of gestational age among neonates born in low-resource settings that illustrates novel clinical and metabolomic parameter-based models. We congratulate the authors for taking up this study on account of several outstanding features:
  - Conceptualizing the study idea of estimating gestational age postnatally, using a mix of conventional and novel metabolomic based objective parameters.
  - Enrolling neonates in large numbers from population based cohort to develop the model as well as validating internally.
  - Stratified random distribution of neonates among the derivation sub-cohorts to match the overall gestational age (GA) of the development cohort.
  - Using efficient study design of retrospective databases to source the samples - often stringently available in neonatal prospective cohort studies.
  - Utilizing machine-learning approaches to refine the algorithm.
  - Undertaking the enormous task of external validation rigorously - involving settings that may find the algorithm most useful, recruiting huge cohorts, harmonizing tools and processes across the sites, etc.
  - Finally, ensuring that the data is accessible to all interested in taking up future studies.

However, we have a few comments to make, especially from the clinical rather than public health viewpoints. Major comments are: the model’s performance in term infants seems more reliable than preterms or SGAs. However the latter are the subgroups where gestation estimation maybe much more useful in clinical settings. One of the reasons could have been because the derivation cohort itself had very few preterm infants (less than 5%) and so future studies should be planned for preterms and SGA categories. Also, as elaborated further, there appears to be much scope for conducting large multi-centric prospective study to remarkably improve and validate the GA algorithm, given that the article presents a promising alternative to grapple with this long-standing issue.

- We are of the opinion that GA estimation by the algorithm beyond ± 1 week may not be clinically as useful; so, the model performance in Table 4 in the main text should primarily depict this parameter and avoid parameters like “% ±  14 d”. The latter may actually be shifted to the web appendix if needed at all. This may also make the table less unsettling to the eyes.
- The authors admit that the model performance got attenuated from derivation to external validation such that the best performing model i. e. #3 lost its accuracy remarkably from average MAE of 0.71 (0.71, 0.72) in Ontario cohort to MAE of 0.90 (0.90, 0.91) and 0.89 (0.86, 0.91) in Manila and Shanghai cohorts respectively. The authors have rightly attributed this finding to factors like biological differences, sample collection or lab/equipment variations and as yet unknown data (pre-)processing techniques.
- However, as depicted in Figure 1 and accepted by the authors, *during external validation* among the different models, the full model (#3) only marginally improved the agreement over that already achieved by birthweight model or model 1 (average MAE 0.90 (Philippine)/0.89 (China) vs 0.96 (Canada)). This is *in contrast to internal validation*; addition of metabolic analytes (i.e. model #3; average MAE 0.71) or for that matter, restricting to analytes only model (model #2; 0.79) had significantly improved over the basic birthweight model (0.96). Two points emerge from this discussion:
  - Firstly, birthweight remains the most fundamental factor to predict GA with the lion’s share of explanatory attribute; this implies possible variability in measuring birthweight, quite plausible in resource-constrained settings and appears to be the crucial factor and should be minimized.
  - Secondly, the addition of analytes may not substantially improve beyond the bithweight’s robust contribution in the algorithm unless we consider critical remedies. One most likely pointer towards the solution may be attributed to the postnatal age cut off taken to collect the samples for metabolomic studies. The Ontario cohort’s sample collection cut off was 48 h compared to the other two cohorts of 72 h. Though the authors do mention that in the latter two cohorts, “most samples would have been excluded” with 48 h cut off, it may be pertinent to give the break up to describe the “most”.
  - Further it will be worthwhile to see how the model performs by removing the samples between 48 h and 72 h, and the same be included in the appendix.
  - If possible, analyzes involving samples more closer to birth e.g. within 24 h should also be alluded to give a broader understanding to the audience. Such exercises may aid towards improving the current performance of the model at nearly 75% of samples being predicted with GA ±  1 w. We do believe certainly that the latter target should be much higher- maybe close to 90%.
  - Thus, as raised earlier, we would like to emphasize that a prospective cohort study design for validation with the samples collected well within 48 h may lead to better algorithm development. In fact, we would suggest this esteemed group of authors led by Hawken *et al.* to consider developing algorithm especially for the preterm infants recruiting subjects prospectively.
- Reference GA assessment for the Philippines cohort has been mentioned to have “generally received gestational dating ultrasounds” for infants born in private hospitals while “other infants GA were generally assessed using Ballard scoring”. This discrepancy probably explains why the Philippines restricted models (#3 or #2) did not perform well while validating with Manila cohort than with Ontario cohort. For example, for model # 3, the average MAE is higher for Manila cohort at 0.90 (0.90, 0.91) against Ontario cohort at 0.72 (0.71, 0.72) (Table 4). In contrast, the average MAE for China restricted model against Ontario cohort (0.76; 0.75, 0.76) is slightly closer to Shanghai cohort (0.89; 0.86, 0.91).
  - Therefore, firstly, we would suggest to provide the break up of two methods of reference GA ascertainment for the Philippines cohort.
  - Secondly, we would like the authors to consider reviewing the analyses of the Manila cohort excluding the infants with GA assessed by Ballard scoring. We assume the remaining cohort may still be large to validate the algorithm robustly given that it originally comprises of over 80,000 infants.
  - In addition, the need for the suggested review may also be relevant because of another example, of SGA cohorts. As highlighted by the authors, model 2 should better perform in the SGA cohorts. However, for Manila SGA10 cohort, model 2 of Philippines restricted model has higher MAE ( **1.08**; 1.06, 1.09) compared to that of model 3 ( **0.97**; 0.96, 0.99). This is contrary to the Canadian scenario: ( **0.90**; 0.85, 0.94) vs. ( **1.13**; 1.09, 1.17) or even the Chinese scenario: ( **1.00**; 0.84, 1.15) vs. ( **1.48**; 1.32, 1.64).
- It will also be worthwhile to have a view at the agreement plots (as in Figure 3) after removing the SGAs - SGA10, SGA3 and both in that order, especially in the Philippine cohort where SGAs constitute around 13% infants. This may also improve the average MAEs across the models 1 – 3. Additionally, separate agreement plots for SGAs should be explored as well, and considered to be included in the appendix if they make sense. This may particularly helpful for deveral LMICs regions having high prevalence of IUGR like South-east Asian Region.
- We have noted a typo error: the proportion of infants predicted by Philippines restricted model for the Ontario cohort within +/- 2 weeks is mentioned as 69.9; this should perhaps be 96.9, going by the 95% CIs.

Is the work clearly and accurately presented and does it cite the current literature?

Yes

If applicable, is the statistical analysis and its interpretation appropriate?

I cannot comment. A qualified statistician is required.

Are all the source data underlying the results available to ensure full reproducibility?

Yes

Is the study design appropriate and is the work technically sound?

Yes

Are the conclusions drawn adequately supported by the results?

Yes

Are sufficient details of methods and analysis provided to allow replication by others?

Yes

Reviewer Expertise:

Neonatology, Neonatal Sepsis, Infectious disease epidemiology

We confirm that we have read this submission and believe that we have an appropriate level of expertise to confirm that it is of an acceptable scientific standard.

# Peer review: Reviewer response for version 1

**DOI:** 10.21956/gatesopenres.14318.r30526

**Published:** 2021-04-26

**Authors:** Julie Courraud

**Affiliations:** 1 Section for Clinical Mass Spectrometry, Statens Serum Institut, Copenhagen, Denmark

The authors report the results of a validation study of previously developed algorithms to predict gestational age in post-natal settings. This is a complex task considering the many differences between the countries and the authors have gathered substantial datasets for this study. One model is based on sex and birthweight only. The two other models include metabolic profiles measured on dried blood spot samples during newborn screening for congenital disorders. The models were applied on data acquired in different laboratories in China and the Philippines in comparison with data from a Canadian laboratory where it was developed. The ultimate goal is legitimate and, although the results are promising, given the limitations observed for preterm infants, several aspects must be addressed and explored before the method can be used in the clinic.

 I could not assess the relevance or correct application of the deep learning method used, as well as bootstrap percentile confidence intervals, as these are not within my area of expertise.

**Comments:**

**Introduction:**

- In paragraph 2, you state that “Samples are typically collected within the first few days after birth, but under special circumstances (e.g., preterm birth, neonatal transfer) may be collected later”. In your study, you selected samples collected within 48 hours only, and explain that the reason is that LMIC usually collect the samples in this timeframe. However, you also state that “most samples would have been excluded if the >48-hour exclusion were applied to these validation cohorts” (Methods, paragraph 2), so it seems that many samples were collected between 48 and 72 hours as mentioned in the discussion.
   
  - Please discuss whether your algorithm could then be used to target the right population, i.e. preterm birth, when the samples might not be collected within 48 or even 72 hours.
  - In untargeted metabolomics of newborn dried blood spots, it has been shown that the baby’s age at sampling is a critical variable when one considers metabolic profiles, and only a few days difference has significant impact. Have you investigated the extent of impact of this variable on your targeted metabolic profiling? How do you intend to address this in your future research or when applying your algorithm? Have you considered integrating age at sampling as a variable in the algorithm (or as a stratification variable for the partitioning into subsets)? See also my comment regarding the discussion.
  - Please discuss the limitation of applying the algorithm **outside** the age at sampling range on which it was developed (or mention/rephrase this limitation more specifically in the discussion: “one day later after birth *than the samples used for model development*” as no sample >48 hours was included during model development).

- In untargeted metabolomics of newborn dried blood spots, another crucial covariate impacting metabolic profiling is month of birth (see Courraud *et al.* 2021), or so at least in Denmark. Being born in summer or winter is remarkably visible. Such effect might be or not be visible in various countries. Have you investigated this potential covariate in your targeted profiling and/or considered integrating it in the algorithm?

**Methods:**

- Paragraphs 3-5. Please specify which analytical methods are used in each center included in the study. Is it mass spectrometry everywhere? Do they use a marketed kit or laboratory-developed tests? Consider giving more methodological details as supplementary material as different platforms may not give the same analytical performance.
- Paragraph 4. Please clarify why some infants get the expanded screening panel of 28 diseases and discuss the risk of selection bias when choosing these infants for the validation.
- Paragraph 5. Please discuss the risk of selection bias when choosing to include only infants for whom tandem mass spectrometry data were available. Does this mean that all metabolites have been measured with this method? (Is this the method used to screen for phenylketonuria, congenital adrenal hyperplasia, hypothyroidism and Glucose-6-phosphate dehydrogenase deficiency?).
- If the applicability of the algorithm is dependent on the family’s income (being able to pay for extra screening), will it achieve its goal to reflect preterm birth globally, given that preterm birth is more frequent in families struggling economically? Please discuss.
- Paragraph 6 on GA assessment: for the Philippines, please indicate the proportion of infants for whom GA was assessed by ultrasound or using Ballard Scoring. A note on the precision of the Ballard scoring with a relevant reference would help the reader.
- On the same topic, you later state that “model performance was assessed by comparing the estimated GA from the model to the ultrasound-derived GA”. So it is unclear whether or not the infants for whom GA was assessed using Ballard Scoring are included at all. Please clarify.

**Table 1:**

- Please indicate what “C0”, “C2”, etc. refer to precisely. It might be obvious for someone in the field, but not to many readers for whom C18 might just be a free fatty acid and not the acyl-carnitine. You could for instance provide a list in supplementary data with full names and PubChemIDs. It helps bridging with the untargeted metabolomics community who is also working on the topic.
- Please be more specific as to which metabolites are included in model 2 and 3. It’s not clear, especially considering the “restricted models” in Table 4.

- Models including newborn screening analytes: How did you cope with the metabolites missing in the validation cohorts? In the result section, you mention “Philippines-restricted” models, etc., please introduce them in the method section. Are the equations the same, just removing the missing metabolites or did you “re-develop” the models? Or?
- Are your models “resistant” to missing values? (In the real world, there will be missing values.)
- Would it be possible to report which metabolites have the biggest influence in each model?
- Have you considered a “model 4” restricted to the few metabolites measured for the “basic” screening panels offered in China and the Philippines? It would be accessible to more people as far as I understand, and might still perform better than just birthweight and sex.
- Statistical modeling: while MAE is clearly explained, it is unclear how RMSE is calculated and what it brings. An extra sentence would be welcome, for instance with an example as given for MAE. RMSE values in Table 4 are not discussed in the manuscript, so if this metric does not bring important elements to understand the work, consider giving the values in supplementary data. Else, please discuss this metric.

**Results and discussion:**

- Can you comment on the high percentage of SGA in the Filipino cohort? Could it be that the thresholds used (ref 14) are not applicable to this population? Could it also be why models generally perform better in the Filipino cohort for the SGA infants as compared to Canadian and Chinese cohorts? (More power).
- In relation to my comment above (introduction), you mention that a majority of Chinese samples have been collected between 48-72 hours. Without going into extensive details, could you present your hypotheses as to why this variable matters? (Are there special metabolic changes during this window for instance?).

**Minor comments:**

**Introduction and methods:**

- Paragraph 3. “in cohorts of infants from in North American settings”. Please remove the “from”.
- Data cleaning and normalization: Please develop the LMIC abbreviation.
- Statistical modeling: “a reported MAE of 1.0 weeks”. Please write “week” for values below 2.0 weeks throughout the manuscript (several places).

**Results:**

- Table 3:
   
  - Females in Canadian cohort, please correct the percentage to 50.7%.
  - Birthweight values. I do not think that the decimal makes sense considering the precision of the measurements. I doubt that the used equipment goes below 1 g of precision. I would present birthweight with no decimal.
  - Please use only one decimal for percentage of SGA counts.
- Please add thousands separators throughout the manuscript in a homogeneous way.
- Internal validation: The second sentence is 61 words long. Please split it in 2-3 sentences for clarity.
- External validation in the Philippines cohort: please indicate the CI for model 1 and 2 regarding the estimated preterm birth rate.
- Same comment for estimated preterm birth rate in the Chinese cohort using model 2.
- Figure 1.
  - (A) It would be more informative to describe models as follows:  Model 1: sex + birth weight; Model 2: sex + analytes; Model 3: sex + birth weight + analytes. “analyte model” and “full model” are not very clear.
  - (C) redundant x axis legend.

**Discussion:**

- You write: “First, as observed in the differences in performance across the birth weight-only models developed in the three cohorts, the predictive utility of anthropomorphic measurements for estimating GA may vary across populations”.
   
  - “birth weight-only models developed ” Do you mean the unique model 1 (sex + birth weight) applied in the 3 cohorts? This sentence is confusing as it implies that there are several models that were developed, when I had understood that you ***developed*** one model 1 based on the Canadian infants and ***applied*** “the final equations” to the other cohorts no involved in the development. Please clarify.
  - Also, why do you think that the “predictive **utility** of anthropomorphic measurements for estimating GA may vary across populations”? It could be that anthropomorphic measurements are indeed too different between Canada and Asian populations, so the models developed with Canadian data are not performing in Chinese infants. But why question the utility of the measurement itself? (To make a comparison with, for instance, month of birth, one could argue that seasonal variation is relevant in some climates but not in others. I’m not sure why birthweight would be more or less relevant and I’m just curious as to whether you have a more specific hypothesis.)
- Sentence starting with “Previous validation of our models among”: Please split this sentence as it is too long and difficult to know what you are referring to when you end with “differences were more pronounced” (between what? These different subgroups? More pronounced compared to?). When you write “inherent biological differences”, do you mean both genetic and environmental? Please clarify.

Is the work clearly and accurately presented and does it cite the current literature?

Yes

If applicable, is the statistical analysis and its interpretation appropriate?

I cannot comment. A qualified statistician is required.

Are all the source data underlying the results available to ensure full reproducibility?

No

Is the study design appropriate and is the work technically sound?

Yes

Are the conclusions drawn adequately supported by the results?

Yes

Are sufficient details of methods and analysis provided to allow replication by others?

Partly

Reviewer Expertise:

Metabolomics, LC-MS/MS, clinical study design, clinical assays and quality

I confirm that I have read this submission and believe that I have an appropriate level of expertise to confirm that it is of an acceptable scientific standard, however I have significant reservations, as outlined above.

# Response

**Published:** 2021-05-27

**Authors:** Kumanan Wilson

**Affiliations:** Ottawa Hospital Research Institute, Canada

**Introduction:**

1. In paragraph 2, you state that “Samples are typically collected within the first few days after birth, but under special circumstances (e.g., preterm birth, neonatal transfer) may be collected later”. In your study, you selected samples collected within 48 hours only, and explain that the reason is that LMIC usually collect the samples in this timeframe. However, you also state that “most samples would have been excluded if the >48-hour exclusion were applied to these validation cohorts” (Methods, paragraph 2), so it seems that many samples were collected between 48 and 72 hours as mentioned in the discussion.

***Response: Thank you for your careful review of the manuscript. We have removed the second half of the sentence as we now see that it could confuse reviewers.***

***We have now provided more details on the exclusion criteria applied to the Ontario cohort for model development. The two criteria leading to the most exclusions were 1) requiring gold-standard GA measurement via 1st-trimester dating ultrasound, and 2) screening bloodspot collection within 48 hours of birth.***

***The first exclusion criteria may have excluded infants born in rural or underserved areas of the province where access to comprehensive prenatal care was lower. In many cases however, this is more likely to be a data quality issue, where dating ultrasound was used but not recorded as such. The second criteria led to the disproportionate exclusion of preterm infants who more often had delayed sample collection, despite this not being recommended practice. Although this exclusion biased the rate of preterm gestation observed in our Ontario study cohort downward, but it was unlikely to have had any important impact on GA model development, as we still had a large sample size across the full spectrum of gestational ages at birth to allow robust model development and performance evaluation. Further, the inclusion of samples collected later than 48 hours would introduce a large amount of heterogeneity in analyte levels which had to be balanced against the impact of exclusions. Although our intention was to exclude samples collected later than 48 hours for the external cohorts as well, it was not possible to take this approach because only calendar day of sample collection was available. Since hourly data was not available, a 48-hour cut off would have excluded most samples. Therefore, we relaxed the exclusion criteria to >72 hours. We have reorganized the methods and provided additional details which will clarify some of these points.***

  

1. Please discuss whether your algorithm could then be used to target the right population, i.e. preterm birth, when the samples might not be collected within 48 or even 72 hours.

***Response: We have reviewed data collected outside of the recommended time frame. In our first external validation study in Bangladesh (Murphy et al, eLife 2019), mean sample collection time was ~14 hours as mothers were often discharged before the 24-hour time frame.  If samples are collected too early, hemoglobin values still reflect maternal values and decreases the accuracy of the algorithm.***

***Ultimately for the algorithm to be viable, it needs to be effective across a range of sample collection timings to accommodate for early or late collection times.***

***For accuracy of newborn screening, it is recommended that samples are collected between 24-48 hours as heterogeneity begins to appear after 48 hours. Limiting the collection time for algorithm development reduces the variability in the data and improves the accuracy of the algorithm.***

 2. In untargeted metabolomics of newborn dried blood spots, it has been shown that the baby’s age at sampling is a critical variable when one considers metabolic profiles, and only a few days difference has significant impact. Have you investigated the extent of impact of this variable on your targeted metabolic profiling? How do you intend to address this in your future research or when applying your algorithm? Have you considered integrating age at sampling as a variable in the algorithm (or as a stratification variable for the partitioning into subsets)? See also my comment regarding the discussion.

***Response: This is an important consideration.  We did incorporate time of sample collection in earlier exploratory models, and though a significant term retained in the model, the effect of time at collection appeared to mostly be the addition of noise/heterogeneity rather than having a monotonic relationship with gestational age that improved model estimates.***

 3. Please discuss the limitation of applying the algorithm **outside** the age at sampling range on which it was developed (or mention/rephrase this limitation more specifically in the discussion: “one day later after birth *than the samples used for model development*” as no sample >48 hours was included during model development).

***Response:*** ***We have made the suggested edit in the discussion as follows:***

 “Another source of bias was that samples in infants born preterm in China, and to a lesser extent the Philippines, were much more likely to be collected later than in term infants, and often after 48-72 hours.  Samples collected beyond 48 hours were more heterogenous than samples collected within 48 hours but excluding these would have excluded an unacceptably large proportion of infants overall, especially preterm infants.  Our compromise approach of relaxing the criteria to exclude samples that were collected later than 72 hours after birth, included more infants at the cost of increased heterogeneity, but even so, still excluded a disproportionate number of preterm infants in the China and Philippines.  A combination of these factors likely contributed to the lower than expected preterm birth rates observed in China and in the Philippines validation cohorts, as well as leading to decreased apparent model performance due to more heterogeneous samples (collected 48-72 hours after birth) being included.”

 4. In untargeted metabolomics of newborn dried blood spots, another crucial covariate impacting metabolic profiling is month of birth (see Courraud *et al.* 2021), or so at least in Denmark. Being born in summer or winter is remarkably visible. Such effect might be or not be visible in various countries. Have you investigated this potential covariate in your targeted profiling and/or considered integrating it in the algorithm?

***Response: Thank you for this comment. We are aware of studies demonstrating seasonable variability in newborn screening outcomes (i.e. Ryckman et al., 2013), and also based on discussions with subject matter experts within our Ontario newborn screening, however the effects noted in Ontario data were small. Despite this, we have actually accounted for seasonality in our data normalization process, in which analyte values were standardized within monthly strata over time, to address assay/reagent/standard changes over time, which had the additional benefit of removing any seasonal bias in analyte measurements within the Ontario, China and Philippines screening data.***

***Reference:***

***Ryckman KK, Berberich SL, Shchelochkov OA, Cook DE, Murray JC. Clinical and environmental influences on metabolic biomarkers collected for newborn screening. Clin Biochem. 2013 Jan;46(1-2):133-8. doi: 10.1016/j.clinbiochem.2012.09.013. Epub 2012 Sep 23. PMID: 23010448; PMCID: PMC3534803.***

**Methods:**

 1.  Paragraphs 3-5. Please specify which analytical methods are used in each center included in the study. Is it mass spectrometry everywhere? Do they use a marketed kit or laboratory-developed tests? Consider giving more methodological details as supplementary material as different platforms may not give the same analytical performance.

***Response: In Ontario, hemoglobin profiles were determined by high performance liquid chromatography; neonatal 17-OHP, and TSH were measured using a PerkinElmer AutoDELFIA® Immunoassays; amino acid and acylcarnitine analysis was performed by tandem mass spectrometry; total TREC copy number was measured by quantitative polymerase chain reaction using a ThermoFisher Scientific Viia 7; biotinidase and galactose-1-phosphate uridyltransferase levels were measured using the Astoria-Pacific SPOTCHECK® Pro system.***

***In the Philippines, a commercial kit is used for the measurement of amino acids, succinylacetone, free carnitine, acylcarnitines and PKU by tandem mass spectrometry (NeoBAse 1, Perkin Elmer). 17OHP and TSH to detect CAH and CH, respectively are detected using a Autodelfia kit by flouroimmunoassay (Perkin Elmer). Screening for G6PDH is done fluorometrically.***

***We were unfortunately not able to confirm the details of analytical methods used in China. Based on published works (Shi et al., 2012), we have surmised the following: phenylalanine was measured fluorometrically to detect phenylketonuria*** ***. In congential hypothyroidism, TSH is quantified by radioimmunoassay (RIA), enzyme linked immunosorbent assay (ELISA) or dissociation-enhanced lanthanide flouroimmunoassay (DELFIA). The remaining tests for the expanded panel use tandem mass spectrometry.***

***Reference:***

***Shi XT, Cai J, Wang YY, Tu WJ, Wang WP, Gong LM et al. Newborn screening for inborn errors of metabolism in mainland china: 30 years of experience. JIMD Rep. 2012;6:79-83. doi: 10.1007/8904_2011_119. Epub 2012 Jan 31. PMID: 23430943; PMCID: PMC3565663.***

 2. Paragraph 4. Please clarify why some infants get the expanded screening panel of 28 diseases and discuss the risk of selection bias when choosing these infants for the validation.

***RESPONSE: Although the newborn screening initiatives are meant to be universal in these populations, some tests were paid for by the families. The expanded panel is now covered by National Health Insurance in the Philippines.  As our study only included samples for which the full panel of analytes was available, this could have contributed to a selection bias towards more affluent families.*** ** ***We have added the following to the discussion:***

 “The preterm birth rate that we estimated in the current cohort (Philippines:4.6%, China: 4.8%) was less than previously estimated (Philippines: 15%, China: 7.1%) (Blencowe et al., 2012). Although newborn screening initiatives are meant to be universal in these populations, some tests are paid for by the families. Considering we only tested samples which the full panel was available this could have contributed to selection bias in our sample population towards more affluent families.”

 Reference:

 Blencowe H, Cousens S, Oestergaard M, Chou D, et al.: National, regional, and worldwide estimates of preterm birth rates in the year 2010 with time trends since 1990 for selected countries: a systematic analysis and implications. *The Lancet*. 2012; **379** (9832): 2162-2172. 

 3.Paragraph 5. Please discuss the risk of selection bias when choosing to include only infants for whom tandem mass spectrometry data were available. Does this mean that all metabolites have been measured with this method? (Is this the method used to screen for phenylketonuria, congenital adrenal hyperplasia, hypothyroidism and Glucose-6-phosphate dehydrogenase deficiency?).

***Response:*** ***The metabolites listed for each site with the expanded panel are listed in Table 1 and in Figure 1.  All metabolites listed were measured at each external site. In Shanghai, tests for phenylketonuria, congenital adrenal hyperplasia, hypothyroidism and Glucose-6-phosphate dehydrogenase deficiency are not measured by tandem mass spectrometry and are publicly funded. The expanded panel of tandem mass spectrometry are strongly recommended but participation is voluntary in both external settings at the time of analysis. There is a risk for selection bias toward a more affluent and medically aware population and we have included this in the manuscript.***

***Reference:***

***Shi XT, Cai J, Wang YY, Tu WJ, Wang WP, Gong LM et al. Newborn screening for inborn errors of metabolism in mainland china: 30 years of experience. JIMD Rep. 2012;6:79-83. doi: 10.1007/8904_2011_119. Epub 2012 Jan 31. PMID: 23430943; PMCID: PMC3565663.***

 4.If the applicability of the algorithm is dependent on the family’s income (being able to pay for extra screening), will it achieve its goal to reflect preterm birth globally, given that preterm birth is more frequent in families struggling economically? Please discuss.

***Response: Universal access and coverage for newborn screening is improving around the world. Since the initiation of this project, newborn screening is now fully funded in the Philippines. The authors agree that if the algorithm is dependent on a family’s ability to pay for screening, this could bias preterm birth rates and is not an ideal scenario for implementation of this approach. However, the approach we are evaluating isn’t intended for implementation in either the Philippines or China - this was an opportunistic use of external cohorts with large retrospective screening databases where we could test our models in different geographic settings with results from local screening labs. Our approach is ultimately targeted to LMICs in Africa and South Asia, based on priorities defined by the Gates foundation assuming the necessary panel of analytes would be collected for the purpose of GA estimation.***

 5. Paragraph 6 on GA assessment: for the Philippines, please indicate the proportion of infants for whom GA was assessed by ultrasound or using Ballard Scoring. A note on the precision of the Ballard scoring with a relevant reference would help the reader.

***Response: Although we knew general practice patterns of gestational dating method used in the Philippines, we did not have individual-level data on what method was used in each individual pregnancy, hence we accepted this as an additional source of validation error which would likely have led to larger MAE/RMSE. This was presented as a limitation in the discussion and we have clarified the description in the methods. It now reads:***

 “In the Philippines cohort, mothers who delivered in private hospitals generally received gestational dating ultrasounds while other infants’ GAs were generally measured using Ballard Scoring, however individual-level data identifying which GA measurement method was used was not available.”

***We also added a statement in the discussion about the precision of the Ballard scoring:***

 “Ballard tends to overestimate gestational age with a wide margin of error, particularly in preterm infants (Lee et al., 2016).”

 Reference:

 Lee AC, Mullany LC, Ladhani K, Uddin J, Mitra D, Ahmed P et al. Validity of Newborn Clinical Assessment to Determine Gestational Age in Bangladesh. Pediatrics. 2016 Jul;138(1):e20153303. doi: 10.1542/peds.2015-3303. Epub 2016 Jun 16. PMID: 27313070; PMCID: PMC4925072.

 6. On the same topic, you later state that “model performance was assessed by comparing the estimated GA from the model to the ultrasound-derived GA”. So it is unclear whether or not the infants for whom GA was assessed using Ballard Scoring are included at all. Please clarify.

***Response: Thank you for pointing this out. We added a qualifying statement to correct this in the methods. The sentence now reads as follows.***

 “For each infant, model performance was assessed by comparing the estimated GA from the model to the ultrasound-derived GA (or ultrasound or Ballard in the Philippines) and calculating validation metrics that reflect the precision of model estimates compared to reference GA values­.”

**Table 1:**

 7. Please indicate what “C0”, “C2”, etc. refer to precisely. It might be obvious for someone in the field, but not to many readers for whom C18 might just be a free fatty acid and not the acyl-carnitine. You could for instance provide a list in supplementary data with full names and PubChemIDs. It helps bridging with the untargeted metabolomics community who is also working on the topic.

***Response: As this journal does not allow supplementary materials, the authors feel that providing the full names of all species would be more detail than is needed. We have added a reference to a previous paper which defines the list more exhaustively. We’ve also added subheadings which identify the ‘C’ species as acylcarntines.***

 8. Please be more specific as to which metabolites are included in model 2 and 3. It’s not clear, especially considering the “restricted models” in Table 4

***Response: We have added in a new figure. (Figure 1) to make it more clear which analytes are used in the restricted models.***

 9. Models including newborn screening analytes: How did you cope with the metabolites missing in the validation cohorts? In the result section, you mention “Philippines-restricted” models, etc., please introduce them in the method section. Are the equations the same, just removing the missing metabolites or did you “re-develop” the models? Or?

***Response: The internal validation of models 1-3 was conducted on an independent test dataset of Ontario infants. Since not all of the predictors available for the Ontario dataset were also available for the external datasets, we tailored the models 2 and 3 to include the maximum number of available predictors in each of the external datasets (which we called ‘restricted models’). The list of analytes available at each site is presented in Table 1.  The tailored models were fit in the Ontario dataset, and validated in the external datasets. We have added a figure to clarify the different models tested. The Methods section now states:***

 “A total of 47 newborn screening analytes, as well as sex, birth weight and multiple birth status, were used in the original Ontario model development. GA at birth (in weeks) determined by first trimester gestational dating ultrasound was the dependent variable. Multiple birth status and a subset of screening analytes were not available in the external cohorts (Table 1). Three main models were developed and evaluated in the Ontario cohort (Table 2). For models 2 and 3, we also developed restricted models including only the covariates available in each of the two external cohorts (Figure 1). Restricted models were trained on the Ontario datasets but deployed in the external cohorts.”

 10. Are your models “resistant” to missing values? (In the real world, there will be missing values.)

***Response:*** ***In the current study the sample size was large enough to exclude any samples with missing values and thus no imputation of missing values was done. In our external validation studies (Murphy et al., 2019, Bota AB et al., 2020 and Hawken et al., 2021) where sample size was limited, we used multiple imputation for missing analyte values. If these models were to be implemented in real-world settings, we would use the same methods to impute missing values.***

***References:***

***Murphy M, Hawken S, Cheng W, Wilson L, Lamoureux M, Henderson M et al. Postnatal gestational age estimation using newborn metabolic profiles: A validation study in Matlab, Bangladesh. Elife. 2019 Mar 19;8. pii: e42627. doi: 10.7554/eLife.42627.***

***Bota AB, Ward V, Hawken S, Wilson LA, Lamoureux M, Ducharme R, et al. Metabolic gestational age assessment in low resource settings: a validation protocol. Gates Open Res. 2021 Jan 28;4:150. doi: 10.12688/gatesopenres.13155.2. PMID: 33501414; PMCID: PMC7801859.***

***Hawken S, Ducharme R, Murphy MSQ, Olibris B, Bota AB, Wilson LA, et al. Development and external validation of machine learning algorithms for postnatal gestational age estimation using clinical data and metabolomic markers. 2021.  BMC Pregnancy and Childbirth (under review).***

 11. Would it be possible to report which metabolites have the biggest influence in each model?

***Response:*** ***Birth weight was the strongest predictor of gestational age overall. Seven analytes were the strongest predictors. Based on partial Spearman correlation analysis there were seven analyte covariates that had distinctly stronger partial correlations with GA compared to all others. These seven analytes in order of strength of partial spearman correlation are: fetal-to-adult hemoglobin ratio, 17-OHP, C4DC, TYR, ALA, C5, and C5DC. These details have been added to the methods.***

 12. Have you considered a “model 4” restricted to the few metabolites measured for the “basic” screening panels offered in China and the Philippines? It would be accessible to more people as far as I understand, and might still perform better than just birthweight and sex.******

***Response: We have previously derived and reported on restricted model results that included 17-hydroxyprogesterone(17OHP), thyroid stimulating hormone (TSH), and fetal/adults hemoglobin, as these are highly predictive analytes that can be cost-effectively analyzed using non MS/MS marketed tests. Although these models were predictive, they were far inferior in performance to models evaluating the full set of expanded screening analytes and didn’t reach a promising level of precision in estimating GA, therefore we haven’t pursued them further.***

 13.Statistical modeling: while MAE is clearly explained, it is unclear how RMSE is calculated and what it brings. An extra sentence would be welcome, for instance with an example as given for MAE. RMSE values in Table 4 are not discussed in the manuscript, so if this metric does not bring important elements to understand the work, consider giving the values in supplementary data. Else, please discuss this metric.

***Response: Thank you.  We have elaborated on the difference between RMSE and MAE in the methods section, and have now provided formulas (please see manuscript text for formulas):***

***“***Model accuracy metrics were based on residual errors: the difference between model-estimated GA and reference GA.  Although mean square error (MSE) is typically the loss function used in maximum-likelihood model fitting for continuous outcomes, it is not necessarily the best metric for assessing average agreement in model validation, as it is based on sum of squared differences, and hence is sensitive to large and small residuals.  Therefore, the primary metric we have presented is the mean absolute error (MAE). MAE is the average of absolute values of residuals (values of the model estimate minus the reference GA) across all observations. MAE reflects the average deviation of the model estimate compared to the reference estimate, expressed in the same units as GA (weeks).

 For completeness, as well as for comparability to other published validations, we also report the square root of the MSE (RMSE). Also known as the standard error of estimation, RMSE is also expressed in the same units as GA (weeks).

 Lower values of both MAE and RMSE reflects more accurate model estimated GA.”

**Results and discussion:**

1. Can you comment on the high percentage of SGA in the Filipino cohort? Could it be that the thresholds used (ref 14) are not applicable to this population? Could it also be why models generally perform better in the Filipino cohort for the SGA infants as compared to Canadian and Chinese cohorts? (More power).

***Response: SGA was previously estimated to be 20.9% in an urban cohort of Filipino infants in the Cebu Longitudinal Health and Nutrition Survey (Jones et al 2008) and 22.5% in a smaller cohort (Blake et al 2016). The INTERGROWTH-21 SGA standards population did not include a Filipino cohort in its population, thus it is possible that this is not an acceptable reference for this particular population. Filipino infants born in Ontario have previously been shown to be at higher risk of being born small as compared to other Asian infants (Batsch et al).***

***References:***

***Jones LL, Griffiths PL, Adair LS, Norris SA, Richter LM, Cameron N. A comparison of the socio-economic determinants of growth retardation in South African and Filipino infants. Public Health Nutr. 2008 Dec;11(12):1220-8. doi: 10.1017/S1368980008002498. Epub 2008 May 8. PMID: 18462561; PMCID: PMC2939971.***

***Blake RA, Park S, Baltazar P, Ayaso EB, Monterde DB, Acosta LP, Olveda RM, Tallo V, Friedman JF. LBW and SGA Impact Longitudinal Growth and Nutritional Status of Filipino Infants. PLoS One. 2016 Jul 21;11(7):e0159461. doi: 10.1371/journal.pone.0159461. PMID: 27441564; PMCID: PMC4956033.***

******

***2. ***In relation to my comment above (introduction), you mention that a majority of Chinese samples have been collected between 48-72 hours. Without going into extensive details, could you present your hypotheses as to why this variable matters? (Are there special metabolic changes during this window for instance?).

***Response: For accuracy of newborn screening, it is recommended that samples are collected between 24-48 hours as samples collected beyond that window have increasingly heterogeneous analyte results influenced by multiple exogenous factors that cannot be statistically adjusted for. Limiting the collection time for algorithm development reduces the variability in the data and improves the accuracy of the algorithm.***

**Minor comments:**

**Introduction and methods:**

 1. Paragraph 3. “in cohorts of infants from in North American settings”. Please remove the “from”.

***Response: Thank you, this has been corrected.***

 2. Data cleaning and normalization: Please develop the LMIC abbreviation.

***Response: Thank you, we have made this edit.***

******

 3. Statistical modeling: “a reported MAE of 1.0 weeks”. Please write “week” for values below 2.0 weeks throughout the manuscript (several places).

***Response: We have edited the manuscript to remove the s after any mention of 1 week. However, the authors think that any decimal number should be presented in plural (ie. 0.96 weeks). We defer to the editors recommendations for guidance on this suggestion.***

**Results:**

 4. Table 3: Females in Canadian cohort, please correct the percentage to 50.7%.

***Response: Thank you, we have corrected this.***

 5. Birthweight values. I do not think that the decimal makes sense considering the precision of the measurements. I doubt that the used equipment goes below 1 g of precision. I would present birthweight with no decimal.

***Response: Thank you, we have corrected this.***

 6. Please use only one decimal for percentage of SGA counts.

***Response: We have made this edit in Table 3, thank you.***

 7. Please add thousands separators throughout the manuscript in a homogeneous way.

***Response:  We have corrected this in the manuscript, thank you.***

 8. Internal validation: The second sentence is 61 words long. Please split it in 2-3 sentences for clarity.

***Response: We have edited this sentence.***

******

***9. ***External validation in the Philippines cohort: please indicate the CI for model 1 and 2 regarding the estimated preterm birth rate.           

***Response:  We have corrected this in the manuscript, thank you.***

 10. Same comment for estimated preterm birth rate in the Chinese cohort using model 2.

***Response:  We have corrected this in the manuscript, thank you.***

 11. Figure 1

  - (A) It would be more informative to describe models as follows:  Model 1: sex + birth weight; Model 2: sex + analytes; Model 3: sex + birth weight + analytes. “analyte model” and “full model” are not very clear.
  - * *(C) redundant x axis legend.

***Response: We have edited the figure and figure legend as suggested.***

**Discussion:**

1. You write: “First, as observed in the differences in performance across the birth weight-only models developed in the three cohorts, the predictive utility of anthropomorphic measurements for estimating GA may vary across populations”.
   
  - “birth weight-only models developed ” Do you mean the unique model 1 (sex + birth weight) applied in the 3 cohorts? This sentence is confusing as it implies that there are several models that were developed, when I had understood that you ***developed*** one model 1 based on the Canadian infants and ***applied*** “the final equations” to the other cohorts no involved in the development. Please clarify.

***Response: We developed 3 models in the Ontario cohort which were applied to the external cohorts. Model 1 was a multivariable regression model including sex, birthweight and their interaction. Model 2 included ELASTIC NET regression model including sex, analytes and pairwise interactions among predictors, whereas model 3 used ELASTIC NET regression model including sex, birth weight, analytes and pairwise interactions among predictors. These were then applied to the external cohorts using analytes available in these settings. To clarify this, we have replaced all references to “birth weight only models” with referenced to Model 1 throughout the manuscript.***

 2. Also, why do you think that the “predictive **utility** of anthropomorphic measurements for estimating GA may vary across populations”? It could be that anthropomorphic measurements are indeed too different between Canada and Asian populations, so the models developed with Canadian data are not performing in Chinese infants. But why question the utility of the measurement itself? (To make a comparison with, for instance, month of birth, one could argue that seasonal variation is relevant in some climates but not in others. I’m not sure why birthweight would be more or less relevant and I’m just curious as to whether you have a more specific hypothesis.)

***RESPONSE: This is an important consideration.  As our intent was to externally validate models derived in Ontario infants, we applied Model 1 based on sex and birthweight model coefficients derived in Ontario infants.  Although birth weight was locally standardized within each of the three cohorts, it is possible that deriving local models in China and the Philippines and then applying them locally would yield better performance.  And we did see evidence of this in previous exploratory modeling we have done.  However our intent here was to deploy pre-trained models to use in estimating GA that don’t require an existing database in each new country that is large enough to derive a robust country-specific model.***

 3. Sentence starting with “Previous validation of our models among”: Please split this sentence as it is too long and difficult to know what you are referring to when you end with “differences were more pronounced” (between what? These different subgroups? More pronounced compared to?). When you write “inherent biological differences”, do you mean both genetic and environmental? Please clarify.

***Response: Thank you we have edited the sentence and it now reads:***

 “Previous validation of our models among infants born in Ontario to landed-immigrant mothers from eight different countries across Asia and North and Sub-Saharan Africa suggested that inherent biological differences may not be a significant contributor to newborn metabolic data and the performance of our algorithms (Hawken et al., 2017). However in an external validation of previously developed GA estimation models in a prospective cohort from South Asia (Murphy et al., 2019), the drop in performance was more pronounced, despite the centralized analysis of samples in the Ontario Newborn Screening lab.”

 References:

 Hawken S, Ducharme R, Murphy MSQ, et al.: Performance of a postnatal metabolic gestational age algorithm: a retrospective validation study among ethnic subgroups in Canada. BMJ Open. 2017;7(9):e015615. 28871012 10.1136/bmjopen-2016-015615 5589017

 Murphy MSQ, Hawken S, Cheng W, et al.: External validation of postnatal gestational age estimation using newborn metabolic profiles in Matlab, Bangladesh. eLife. 2019;8:e42627. 30887951 10.7554/eLife.42627 6424558

# Peer review: Reviewer response for version 1

**DOI:** 10.21956/gatesopenres.14318.r30282

**Published:** 2021-03-29

**Authors:** José Villar, Eric Ohuma

**Affiliations:** 1 Nuffield Department of Women's and Reproductive Health, University of Oxford, Oxford, UK; 2 London School of Hygiene & Tropical Medicine, London, UK

In this manuscript by Hawken *et al.*, the authors have performed an external validation of newborn metabolomic markers for postnatal GA estimation in East and South-East Asian infants using an Elastic Net regression modelling approach.

**Main comments:**

- There is no doubt that an accurate estimate of GA is key. However, the authors propose a postnatal estimation of GA which does very little in advancing and encouraging determination of GA in early pregnancy. This is the recommendation of the WHO, The Brighton Collaboration GAIA definitions Prematurity and assessment of gestational age, The National Institute for Health and Care Excellence (NICE) Guideline for Routine Antenatal Care (2008), and International Society of Ultrasound in Obstetrics and Gynaecology (ISUOG).
- There is a clear gap identified in the accuracy of determining GA especially in LMIC and I strongly doubt this approach will help in better characterising of the burden of vulnerable newborns and the potential impact towards achieving better estimates for population rates of preterm birth, low birth weight, small-for-gestational age, and combinations of these to identify other vulnerable newborn phenotypes. 

- Overall reporting of results warrants improvement, the authors have decided to report overall mean agreement in GA between gold standard GA with model predicted GA and yet there are clearly large differences in precision and this differs according to GA (Figure 1). The authors should state explicitly what is meant by agreement within 7 days. For example, if on average, model estimate agrees within 7 days, this technically means ± 7 days and therefore for a given fetus say model GA estimate is 32 weeks + 0 days, this would mean that the true GA ranges between 31 weeks + 0 days and 33 weeks + 0 days which is effectively 2 weeks. Following this, the best model estimate (model 3) on average will be accurate to within 10 days at best. The authors should show a plot of true GA vs. predicted GA as this will evidently show the variability of the prediction as a function of GA as opposed to the aggregated estimates they have presented by GA in Figure 1.

- Across all models, great discrepancies and perhaps unacceptable discrepancies are observed for GA before 39 weeks. I am not convinced this approach offers any benefit/added value/utility compared to other methods in common use such as best obstetric methods for ascertaining GA.

- The team used blood spot samples collected within 48hrs of delivery – there is considerable extra effort involved, time, and cost for drawing blood spots and processing of analytes. I do not see how this would be a feasible alternative especially for LMIC where accurate estimation of GA is a key data gap. The merits of the proposed approach have to clearly outweigh the performance of other known methods for postnatal GA determination such as the Ballard Score.

**Statistical modelling:**

- Statistical modelling uses split-sampling for model derivation and model validation. Data splitting is an unstable method for validating models because if you were to split the data again, develop a new model on the training sample, and test it on the holdout sample, the results are likely to vary significantly. Recommended resampling approaches are cross-validation and bootstrapping and the authors should consider this.
- The authors should also report recommended metrics for evaluating model performance i.e., discrimination and calibration of the models.

- Interaction terms for models 1-3 – could the authors comment on the added value of the interaction parameters and how much improvement in model performance can be attributed to the inclusion of the interaction parameters?
- Reference for classifying SGA – the authors provide the reference INTERGROWTH-21st very preterm size at birth reference charts. The reference provided is only for infants born 24 to <33 weeks. What about for infants born after 33 weeks? Can the authors confirm that for infants born ³33 weeks they used the IG standards provided here: Villar *et al.* (2014 ^1^).

**Results:**

- The authors should comment on the very low % preterm across the three cohorts. According to Blencowe *et al. *(2012 ^2^), in 2010, the preterm birth rate in Philippines was estimated to be 15% (vs 4.6% in current cohort) and was 7.1% in China (vs 4.8% in current cohort).

- In table 3, can the authors also include % low birth weight and % LGA?

**Minor comments:**

- It is unnecessary to have elastic net in the title – it distracts the main focus of the paper.

Is the work clearly and accurately presented and does it cite the current literature?

Yes

If applicable, is the statistical analysis and its interpretation appropriate?

Partly

Are all the source data underlying the results available to ensure full reproducibility?

Yes

Is the study design appropriate and is the work technically sound?

Yes

Are the conclusions drawn adequately supported by the results?

Partly

Are sufficient details of methods and analysis provided to allow replication by others?

Yes

Reviewer Expertise:

Medical Statistician with experience in the field of maternal and child health

We confirm that we have read this submission and believe that we have an appropriate level of expertise to confirm that it is of an acceptable scientific standard, however we have significant reservations, as outlined above.

## References

[1] : International standards for newborn weight, length, and head circumference by gestational age and sex: the Newborn Cross-Sectional Study of the INTERGROWTH-21st Project. *The Lancet*.2014;384(9946) : 10.1016/S0140-6736(14)60932-6 857-868 10.1016/S0140-6736(14)60932-6 25209487

[2] : National, regional, and worldwide estimates of preterm birth rates in the year 2010 with time trends since 1990 for selected countries: a systematic analysis and implications. *The Lancet*.2012;379(9832) : 10.1016/S0140-6736(12)60820-4 2162-2172 10.1016/S0140-6736(12)60820-4 22682464

# Response

**Published:** 2021-05-13

**Authors:** Kumanan Wilson

**Affiliations:** Ottawa Hospital Research Institute, Canada

*Thank you for your review. We have carefully responded to your concerns. We are updating the manuscript accordingly and it will be posted shortly.*

**Reviewer:**

 In this manuscript by Hawken *et al.*, the authors have performed an external validation of newborn metabolomic markers for postnatal GA estimation in East and South-East Asian infants using an Elastic Net regression modelling approach.

**Main comments:**

- There is no doubt that an accurate estimate of GA is key. However, the authors propose a postnatal estimation of GA which does very little in advancing and encouraging determination of GA in early pregnancy. This is the recommendation of the WHO, The Brighton Collaboration GAIA definitions Prematurity and assessment of gestational age, The National Institute for Health and Care Excellence (NICE) Guideline for Routine Antenatal Care (2008), and International Society of Ultrasound in Obstetrics and Gynaecology (ISUOG).

***Response:***

***While the authors agree that accurate estimation of GA in early pregnancy is critical as the reviewer has outlined above, our objective was to provide a non-invasive alternative in low resource settings where prenatal care involving GA dating ultrasound in the first trimester is not widely accessible.***

**Reviewer:**

 There is a clear gap identified in the accuracy of determining GA especially in LMIC and I strongly doubt this approach will help in better characterising of the burden of vulnerable newborns and the potential impact towards achieving better estimates for population rates of preterm birth, low birth weight, small-for-gestational age, and combinations of these to identify other vulnerable newborn phenotypes. 

***Response:***

***Post-natal estimation of GA has been identified as a priority of the Gates Foundation to facilitate population-based surveillance https://www.linkedin.com/pulse/innovation-how-50-year-old-drop-blood-helps-solve-urgent-mundel/ . In many LMIC's, accurate early pregnancy estimations of GA are not accessible due to lack of ultrasound, pre-natal care and the unreliability of LMP. Our goal was to develop and refine one more potential tool to support maternal newborn care and surveillance in low resource settings. This external validation study has provided important information on the strengths and limitations of applying this method in different settings. In combination with the work of others, this may be a component of a broader solution that combines the strengths of different approaches. This may be an acceptable approach for a demographic surveillance site, for example. Ultimately, non-invasive ‘omics methods may provide an alternative to first trimester ultrasound when not available. Furthermore, this approach could assist in distinguishing between SGA infants and pre-term infants to facilitate care at the individual infant level. However, the limitations of these approaches need to be recognized and our work identifies some of these challenges.***

****

**Reviewer:**

 Overall reporting of results warrants improvement, the authors have decided to report overall mean agreement in GA between gold standard GA with model predicted GA and yet there are clearly large differences in precision and this differs according to GA (Figure 1). The authors should state explicitly what is meant by agreement within 7 days. For example, if on average, model estimate agrees within 7 days, this technically means ± 7 days and therefore for a given fetus say model GA estimate is 32 weeks + 0 days, this would mean that the true GA ranges between 31 weeks + 0 days and 33 weeks + 0 days which is effectively 2 weeks. Following this, the best model estimate (model 3) on average will be accurate to within 10 days at best. The authors should show a plot of true GA vs. predicted GA as this will evidently show the variability of the prediction as a function of GA as opposed to the aggregated estimates they have presented by GA in Figure 1.

***Response:***

***Thank you. We have clarified that “within” indicates ± X days from the true GA throughout the manuscript. We have now also included*** ***residual plots of predicted – observed by ultrasound-assigned gestational age (new Figure 2 in*** ***manuscript*** ***).***

**Reviewer:**

 Across all models, great discrepancies and perhaps unacceptable discrepancies are observed for GA before 39 weeks. I am not convinced this approach offers any benefit/added value/utility compared to other methods in common use such as best obstetric methods for ascertaining GA.

***Response:***

***We agree that the model did not perform well in infants born before 39 weeks’ gestation. However, results from our prospective validation studies are much more promising (1,2). In our previous prospective Bangladesh cohort, the model accurately estimated gestational age +/- 6 days (2). The current paper represents one component of our external validation strategy that included: a) validation in retrospective cohorts from established newborn screening programs and b) prospective validation in low-resource settings with primary data collection and better control over the quality of the ascertainment of the gold standard GA measurement.***

  

1. ***Murphy M, Hawken S, Cheng W, Wilson L, Lamoureux M, Henderson M et al. Postnatal gestational age estimation using newborn metabolic profiles: A validation study in Matlab, Bangladesh. Elife. 2019 Mar 19;8. pii: e42627. doi: 10.7554/eLife.42627.***

 

1. ***Hawken S, Ducharme R, Murphy MSQ, Olibris B, Bota AB, Wilson LA, et al. Development and external validation of machine learning algorithms for postnatal gestational age estimation using clinical data and metabolomic markers. BMC Medical Informatics and Decision Making (under review). Preprint: medRxiv 2020.07.21.20158196; doi: https://doi.org/10.1101/2020.07.21.20158196 ***

***The purpose of this study was to evaluate the utility of a model derived in the Ontario dataset but deployed in an external setting. It is possible that deriving models for each external site using the external data may yield more robust results, and this is something to possibly explore in the future.***

 Reviewer:

 The team used blood spot samples collected within 48hrs of delivery – there is considerable extra effort involved, time, and cost for drawing blood spots and processing of analytes. I do not see how this would be a feasible alternative especially for LMIC where accurate estimation of GA is a key data gap. The merits of the proposed approach have to clearly outweigh the performance of other known methods for postnatal GA determination such as the Ballard Score.

***Response:***

***Our objective in this external validation study was not to address feasibility, but rather to assess the performance of models developed in a North American cohort in infants in other international settings. The BMGF has funded our group to assess the feasibility of implementing our GA estimation method in multiple LMIC settings, including comparative accuracy/costs/burden versus other available methods so we will be able to address these important considerations when we publish.***

****

**Statistical modelling:**

**Reviewer:**

 tatistical modelling uses split-sampling for model derivation and model validation. Data splitting is an unstable method for validating models because if you were to split the data again, develop a new model on the training sample, and test it on the holdout sample, the results are likely to vary significantly. Recommended resampling approaches are cross-validation and bootstrapping and the authors should consider this.

***Response:***

***We agree with this comment, but in the context of much smaller databases. Although our modeling process did use bootstrapping and cross-validation in the model training phase (i.e. for optimizing ELASTICNET hyperparameters), our available sample was large enough to ensure that the test subset was large, stable and had a similar distribution of perinatal characteristics. Using a held-out test set also allowed us to exactly replicate the analysis pipeline that would be used in our external validations (i.e. data preprocessing, normalization etc. were executed separately, but using the same algorithm in the model training subset and in the internal validation subset, as well as in external validation settings, which would not be possible if a cross-validation approach was used). To reinforce this point, we conducted sensitivity analyses where we used different training and testing splits, and also a cross-validation approach, and these yielded nearly identical results.***

******

***Reviewer: ***The authors should also report recommended metrics for evaluating model performance i.e., discrimination and calibration of the models.

***Response:***

***In this paper, we did not report results from a logistic regression model or other method meant to classify term vs. preterm birth, so discrimination is not relevant in the context of the models we are reporting. However, based on our model estimates, we have reported the preterm birth rate that is based on observed GA above and below 37 weeks and model-predicted GA above and below 37 weeks. This represents calibration in the large, which we have now clarified in the manuscript, and we have included plots of observed vs predicted GA (new Figure 2 in manuscript).***

**Reviewer:**

 Interaction terms for models 1-3 – could the authors comment on the added value of the interaction parameters and how much improvement in model performance can be attributed to the inclusion of the interaction parameters?

***Response:***

***The inclusion of the interaction terms improved model performance appreciably, both overall and in important subgroups (<37 weeks and SGA10). The addition of the interaction terms reduced the MAE from 0.75 to 0.71 overall, from 1.14 to 1.03 in the <37 weeks subgroup, and from 1.39 to 1.13 in the SGA10 subgroup. We have now commented on the effect of the interactions in the methods section.***

**Reviewer:**

 Reference for classifying SGA – the authors provide the reference INTERGROWTH-21st very preterm size at birth reference charts. The reference provided is only for infants born 24 to <33 weeks. What about for infants born after 33 weeks? Can the authors confirm that for infants born ³33 weeks they used the IG standards provided here: Villar *et al.* (2014 ^1^).

***Response:***

***Yes, we confirm that the reference provided by the reviewer was used for infants born 33 weeks and older. We have updated the text accordingly.***

****

**Results:**

**Reviewer:**

 The authors should comment on the very low % preterm across the three cohorts. According to Blencowe *et al. *(2012 ^2^), in 2010, the preterm birth rate in Philippines was estimated to be 15% (vs 4.6% in current cohort) and was 7.1% in China (vs 4.8% in current cohort). *Thank you we have commented on this in the discussion. The text now reads:*

***Response:*** ***The preterm birth rate that we estimated in the current cohort (Philippines: 4.6%, China: 4.8%) was less than previously estimated (Philippines: 15%, China: 7.1%)(3). Although newborn screening initiatives are meant to be universal in these populations, some tests were paid for by the families. In the Philippines, all newborn screening has been covered since 2019, but prior to that only 4 of the tests were covered. Considering we only tested samples which the full panel was available this could have contributed to selection bias in our sample population where infants born in higher resource/urban areas were preferentially included where preterm birth rates could be substantially different. We also excluded infants in whom samples were collected later than 72 hours after birth as these are subject to a high level of heterogeneity. This had a similar effect as it did in Ontario, disproportionately excluding preterm infants, lowering the preterm birth rate from 6.9% to 4.8% in the China cohort (and thus accounting for the bulk of the discrepancy there) and from 5.6% to 4.62% in the Philippines cohort (thus accounting for only a very small part of the discrepancy).***

 1. Blencowe H, Cousens S, Oestergaard M, Chou D, et al.: National, regional, and worldwide estimates of preterm birth rates in the year 2010 with time trends since 1990 for selected countries: a systematic analysis and implications. *The Lancet*. 2012; **379** (9832): 2162-2172 

**Reviewer:**

 In table 3, can the authors also include % low birth weight and % LGA?

**Response:**

***We have added low birthweight and LGA to Table 3.***

****

**Reviewer;**

**Minor comments:**

- It is unnecessary to have elastic net in the title – it distracts the main focus of the paper.

**Response:**

**Thank you, we have made this change. The title is now: *External validation of machine learning models including newborn metabolomic markers for postnatal gestational age estimation in East and South-East Asian infants*** **

**Reviewer References**

 1. Villar J, Ismail L, Victora C, Ohuma E, et al.: International standards for newborn weight, length, and head circumference by gestational age and sex: the Newborn Cross-Sectional Study of the INTERGROWTH-21st Project. *The Lancet*. 2014; **384** (9946): 857-868 Publisher Full Text

 2. Blencowe H, Cousens S, Oestergaard M, Chou D, et al.: National, regional, and worldwide estimates of preterm birth rates in the year 2010 with time trends since 1990 for selected countries: a systematic analysis and implications. *The Lancet*. 2012; **379** (9832): 2162-2172 Publisher Full Text

# Peer review: Reviewer response for version 1

**DOI:** 10.21956/gatesopenres.14318.r30362

**Published:** 2021-03-26

**Authors:** Sunil Sazawal

**Affiliations:** 1 Centre for Public Health Kinetics, New Delhi, Delhi, India; 2 Johns Hopkins School of Public health, Baltimore, MD, USA

**General Comments:**

 This is an interesting study where the authors have collected unique datasets both from developed country and developing country settings. The paper addresses a very pertinent question related to the gestational age dating especially in the developing country settings where early ultrasound dating is missing due to unavailability of resources or because of the cost involved in getting an ultrasound done. But it is yet to be seen whether taking blood spots in on Whatman paper is feasible and getting the screen done in a lab with MS is possible in these settings. The paper is generally well written and structured with enough details, however, there are some queries which the authors need to address to make the paper clearer and more transparent.

 Paper should be accepted for indexing with revisions.

1. Major Concern:
  - Although not highlighted but proved in the original manuscript there were exclusions and imputations. A section providing details of these and how these may have affected the outcome or made improvements needs to be clearly stated in methods and discussion.
  - The full model seems to include addition Hb ratio’s and analyses not part of the newborn screening routinely, in terms of implications and discussion this needs better discussed. While in results a result that was obtained with metabolic screen with clinical variable routinely available as birth weight needs to be the key primary estimate and other estimates need to be provided as secondary exploratory results. The distinction as provided current is blurred and confuses the reader.
2. Other concerns:
  - Reference GA assessment

*Statement*: In the Philippines cohort, mothers who delivered in private hospitals generally received gestational dating ultrasounds while other infants’ GAs were generally measured using Ballard Scoring

*Q*: It is a discrepancy since Ontario based models trained data against USG confirmed GA. Then under **External validation** where Philippines samples were used: How can both ultrasound and Ballard scoring used under same bracket.

 Internal validation of model performance in Ontario, Canada 

*Q*: Was the internal validation performed with previously developed models including 47 analytes, birth weight, sex or the restricted model, needs clarification and discussed 

*Q*: Restricted model definition

 The proper definition of the restricted model is missing. Was a restricted model built separately for Manila and Shanghai or Separate models model were made for Manila and Shanghai

 Statistical methods

*Statement*: In the Ontario cohort, all screen-positive results were excluded from analysis, which had the effect of removing a large proportion of extreme outliers and a typical metabolic profiles.

*Q*: What is the meaning of screen positive? Does this mean that all children who had a metabolic disorder which might have abnormal values for some metabolites were removed? If so, was it done in the other two datasets and will the model then be not applicable to children who show abnormal values for the metabolites.

Is the work clearly and accurately presented and does it cite the current literature?

Yes

If applicable, is the statistical analysis and its interpretation appropriate?

Yes

Are all the source data underlying the results available to ensure full reproducibility?

No

Is the study design appropriate and is the work technically sound?

Yes

Are the conclusions drawn adequately supported by the results?

Partly

Are sufficient details of methods and analysis provided to allow replication by others?

Partly

Reviewer Expertise:

Infectious disease, pediatrics, epidemiology and statistics, global health

I confirm that I have read this submission and believe that I have an appropriate level of expertise to confirm that it is of an acceptable scientific standard, however I have significant reservations, as outlined above.

# Response

**Published:** 2021-05-05

**Authors:** Kumanan Wilson

**Affiliations:** Ottawa Hospital Research Institute, Canada

**Reviewer 1** 

 General Comments: 

 This is an interesting study where the authors have collected unique datasets both from developed country and developing country settings. The paper addresses a very pertinent question related to the gestational age dating especially in the developing country settings where early ultrasound dating is missing due to unavailability of resources or because of the cost involved in getting an ultrasound done. But it is yet to be seen whether taking blood spots in on Whatman paper is feasible and getting the screen done in a lab with MS is possible in these settings. The paper is generally well written and structured with enough details, however, there are some queries which the authors need to address to make the paper clearer and more transparent. 

 Paper should be accepted for indexing with revisions. 

 Major Concerns: 

- Although not highlighted but proved in the original manuscript there were exclusions and imputations. A section providing details of these and how these may have affected the outcome or made improvements needs to be clearly stated in methods and discussion 

 

**Response:** *No imputation of missing analyte/covariate values was undertaken either in the Ontario cohort or in the international cohorts given large sample sizes and very low occurrence of missing values. We have now provided further detail on the exclusion criteria applied in preparing the Ontario cohort for model development. The two criteria leading to the most exclusions were 1) requiring gold-standard GA measurement via 1st-trimester dating ultrasound, and 2) screening bloodspot collection within 48 hours of birth.*

*The first exclusion criteria may have excluded infants born in rural or underserved areas of the province where access to comprehensive prenatal care was lower. In many cases however, this is more likely to be a data quality issue, where dating ultrasound was used but not recorded as such. The second criteria led to the disproportionate exclusion of preterm infants who more often had delayed sample collection, despite this not being recommended practice. Although this exclusion biased the rate of preterm gestation observed in our Ontario study cohort downward, but it was unlikely to have had any important impact on GA model development, as we still had a large sample size across the full spectrum of gestational ages at birth to allow robust model development and performance evaluation. Further, the inclusion of samples collected later than 48 hours would introduce a large amount of heterogeneity in analyte levels which had to be balanced against the impact of exclusions. We have added more details to the methods and discussion reflecting these considerations.*

**

   

**Reviewer:**

 The full model seems to include addition Hb ratio’s and analyses not part of the newborn screening routinely, in terms of implications and discussion this needs better discussed. While in results a result that was obtained with metabolic screen with clinical variable routinely available as birth weight needs to be the key primary estimate and other estimates need to be provided as secondary exploratory results. The distinction as provided current is blurred and confuses the reader. 

**Response:**

*Hemoglobin types (eg HbF HbA - fetal and adult hemoglobin types) are measured during routine NBS in Ontario and the Philippines, in the course of identifying mutant types associated with hemoglobinopathies. These are reported as “peak percentages” with respect to total hemoglobin.  We have used the peak percentages for normal HbF, HbF1 and HbA to construct a ratio of fetal to adult hemoglobin by calculating (HbF+HbF1)/(HbF+HbF1+HbA), to measure the proportion of normal fetal hemoglobin, relative to the proportion of total normal fetal + adult hemoglobin types. This is strongly predictive of gestational age as the transition from fetal to adult hemoglobin occurs apace with fetal development. We have added these details to the Methods.*

   

**Reviewer:**

 Other concerns: 

 Reference GA assessment 

 Statement: In the Philippines cohort, mothers who delivered in private hospitals generally received gestational dating ultrasounds while other infants’ GAs were generally measured using Ballard Scoring 

 Q: It is a discrepancy since Ontario based models trained data against USG confirmed GA. Then under External validation where Philippines samples were used: How can both ultrasound and Ballard scoring used under same bracket. 

**Response:**

*Although we knew general practice patterns of gestational dating method used in the Philippines, we did not have individual-level data on what method was used in each individual pregnancy, hence we accepted this as an additional source of validation error which would lead to larger MAE/RMSE. This was presented as a limitation in the discussion and we have clarified the description in the methods. It now reads:*

*‘In the Philippines cohort, mothers who delivered in private hospitals generally received gestational dating ultrasounds while other infants’ GAs were generally measured using Ballard Scoring, however individual-level data identifying which GA measurement method was used was not available.’*

**Reviewer:** 

 Internal validation of model performance in Ontario, Canada  

 Q: Was the internal validation performed with previously developed models including 47 analytes, birth weight, sex or the restricted model, needs clarification and discussed  

**Response:**

*The internal validation of models 1-3 was conducted on an independent test dataset of Ontario infants using all 47 analytes, multiple gestation, birthweight and sex. Since not all of the predictors available for the Ontario dataset were also available for the external datasets (multiple gestation and a small subset of analytes were absent), we tailored the models to include the maximum number of available predictors in each of the external datasets (which we called ‘restricted models’). The list of analytes available at each site is presented in Table 1. The tailored models were fit in the Ontario dataset, and validated in the Ontario test set and external datasets. We have added a figure (Figure 1) to clarify the different models tested. The Methods section now states:*

**

*'A total of 47 newborn screening analytes, as well as sex, birth weight and multiple birth status, were used in the original Ontario model development. GA at birth (in weeks) determined by first trimester gestational dating ultrasound was the dependent variable. A subset of screening analytes, as well as multiple gestation status were not available in the external cohorts (Table 1). Three main models were developed and evaluated in the Ontario cohort (Table 2). For models 2 and 3, we also developed restricted models including only the covariates available in each of the two external cohorts (Figure 1). Restricted models were trained on the Ontario datasets but deployed in the external cohorts.'*

**Reviewer:**

 Q: Restricted model definition 

 The proper definition of the restricted model is missing. Was a restricted model built separately for Manila and Shanghai or Separate models model were made for Manila and Shanghai 

**Response:**

*Restricted models were built separately to be applied in Manila and Shanghai based on availability of screening analytes/predictors in each setting. These models were trained in the Ontario data and then deployed in the external cohorts. We have updated the text and the methods now read:*

**

*A total of 47 newborn screening analytes, as well as sex, birth weight and multiple birth status, were used in the original Ontario model development. GA at birth (in weeks) determined by first trimester gestational dating ultrasound was the dependent variable. A subset of screening analytes as well as multiple gestation were not available in the external cohorts (Table 1). Three main models were developed and evaluated in the Ontario cohort (Table 2). Model 1 was developed excluding multiple gestation status, and for models 2 and 3, we also developed restricted models including only the covariates available in each of the two external cohorts (Figure 1). All of the restricted models were trained on the Ontario datasets and deployed in the external cohorts.*

**Reviewer:**

 Statistical methods 

 Statement: In the Ontario cohort, all screen-positive results were excluded from analysis, which had the effect of removing a large proportion of extreme outliers and atypical metabolic profiles. 

 Q: What is the meaning of screen positive? Does this mean that all children who had a metabolic disorder which might have abnormal values for some metabolites were removed? If so, was it done in the other two datasets and will the model then be not applicable to children who show abnormal values for the metabolites. 

**Response:** 

*Screen positive refers to infants who tested positive for a disorder in the screening panel. We have clarified this statement in the methods. These infants were excluded from the Ontario population as they tend to have extreme outlying values for some analytes which impact negatively on model development. Additionally, we employed a strategy of winsorizing extreme values that lay more than three IQRs above the third quartile or three IQRs below the first quartile. Winsorizing replaces these extreme outliers with the upper and lower boundary value for the analyte, which preserves the extremeness, but reduces the impact of the original value. The same winsorization algorithm was applied in the external cohorts. Screen positive data points were not explicitly removed from the Philippines and China datasets. The reviewer is correct that the model may not be as accurate for children with abnormal values, however in the external settings where this algorithm is being deployed, it would not be known whether infants had a disorder at birth, so the model would need to be as robust as possible in estimating GA under these conditions. Because of the approach we took, the impact of abnormal/extreme values would be attenuated by our data normalization strategy which included both log transformation and Winsorization of extreme outliers. The occurrence of extreme outliers for either screen positive infants or for other reasons was extremely low, so would only affect a small number infants, but our strategy allowed us to produce a GA estimate in these infants that was robust to extreme values and less likely to produce a wildly inaccurate estimate. We have clarified these details in the Methods.*
